# Regulation of GSTu1-mediated insecticide resistance in *Plutella xylostella* by miRNA and lncRNA

**DOI:** 10.1371/journal.pgen.1009888

**Published:** 2021-10-28

**Authors:** Bin Zhu, Linhong Li, Rui Wei, Pei Liang, Xiwu Gao

**Affiliations:** Department of Entomology, China Agricultural University, Beijing, China; University of Kentucky, UNITED STATES

## Abstract

The evolution of resistance to insecticides is well known to be closely associated with the overexpression of detoxifying enzymes. Although the role of glutathione *S*-transferase (GST) genes in insecticide resistance has been widely reported, the underlying regulatory mechanisms are poorly understood. Here, one GST gene (*GSTu1*) and its antisense transcript (lnc-*GSTu1*-AS) were identified and cloned, and both of them were upregulated in several chlorantraniliprole-resistant *Plutella xylostella* populations. GSTu1 was confirmed to be involved in chlorantraniliprole resistance by direct degradation of this insecticide. Furthermore, we demonstrated that lnc-*GSTu1*-AS interacted with *GSTu1* by forming an RNA duplex, which masked the binding site of miR-8525-5p at the *GSTu1*-3′UTR. In summary, we revealed that lnc-*GSTu1*-AS maintained the mRNA stability of *GSTu1* by preventing its degradation that could have been induced by miR-8525-5p and thus increased the resistance of *P*. *xylostella* to chlorantraniliprole. Our findings reveal a new noncoding RNA-mediated pathway that regulates the expression of detoxifying enzymes in insecticide-resistant insects and offer opportunities for the further understanding of the mechanisms of insecticide and drug resistance.

## Author summary

The development of insecticide resistance in insect pests is a worldwide concern and a major problem in agriculture. Understanding the genetics of insecticide resistance is critical for effective crop protection. *Plutella*. *xylostella* (L.), a major pest of cruciferous crops, has developed resistance to almost all kinds of insecticide, and has become one of the most resistant pests in the world. Overexpression of detoxification enzymes is closely associated with insecticide resistance, but researches on their regulatory mechanism are still very limited. Here, *GSTu1* was identified to be upregulated in several chlorantraniliprole-resistant *P*. *xylostella* populations and was confirmed to be involved in chlorantraniliprole resistance by direct degradation of this insecticide. Further, lnc-*GSTu1*-AS transcribed from the opposite DNA strand to *GSTu1* was identified to be able to enhance the mRNA stability of *GSTu1* by blocking miRNA activity, and thus increased the resistance of *P*. *xylostella* to chlorantraniliprole. The results provide further insights into the mechanisms underlying metabolic resistance.

## Introduction

Insect pests cause serious damage to the crop yield directly and indirectly, which costs billions of dollars annually [1]. Although awareness of the importance of integrated pest management (IPM) is growing, the use of chemical insecticides is still the main strategy for controlling insect pests [2, 3]. However, the control efficacy is threatened by the evolution of insecticide resistance, which poses a continuing influence on agricultural production, environmental safety and even human health [4, 5]. The diamondback moth, *Plutella*. *xylostella* (L.), a major pest of cruciferous crops, is one of the most resistant insects globally [6, 7]. Chlorantraniliprole is an anthranilic diamide insecticide that shows remarkable efficacy in the control of several orders of insect pests, especially lepidopteran pests, but has low toxicity towards untargeted organisms [8, 9]. However, in recent years, *P*. *xylostella* has developed high-level resistance to chlorantraniliprole in many countries [10–12].

Amino acid mutations (G4946E and I4790M/K) in the ryanodine receptor (the target of diamide insecticides) have been considered one of the main causes of chlorantraniliprole resistance [13–18]. The upregulation of detoxification enzymes or xenobiotic transporters is also reported to play important roles in diamide insecticide resistance. To date, several detoxification enzyme genes, including two cytochrome P450 genes (*CYP6BG1* and *CYP321E1*) [19, 20], one uridine diphosphate-glycosyltransferase (UGT) gene (*UGT33AA4*) [21] and one flavin-dependent monooxgenase (FMO) gene [22], have been found to play a role in chlorantraniliprole resistance in *P*. *xylostella*.

Metabolic detoxification is very important in insecticide resistance [23, 24]. In addition to P450, FMO and UGT, glutathione *S*-transferases (GSTs) may also be involved in chlorantraniliprole resistance. For example, the activity of GSTs was significantly increased 24 h post chlorantraniliprole exposure in *Bombyx mori* [25]. Additionally, RNA-seq analysis showed that three GST genes were differentially expressed with increases in resistance to chlorantraniliprole in *P*. *xylostella* [26]. In our previous study, RNA-seq analysis showed that one GST gene (named *GSTu1* in this study) was upregulated in a chlorantraniliprole-resistant *P*. *xylostella* population. Interestingly, another transcript (predicted as an antisense long noncoding RNA, lncRNA) that had generic exonic overlap with *GSTu1* on the opposite strand was also upregulated [27]. Thus, we inferred that these two transcripts might play a role in chlorantraniliprole resistance in *P*. *xylostella*.

Noncoding RNAs (ncRNAs) are functional RNA molecules that are not translated into proteins and are usually categorized into two main groups: small and long ncRNAs [28, 29]. The focus of this study was mainly on microRNAs (miRNAs) and lncRNAs. MiRNAs are short (∼22 nt), endogenous, noncoding RNAs, which regulate gene expression mainly through binding to the 3’-untranslated region (3’-UTR) of target RNA transcripts and eventually cause cleavage, translational repression or mRNA decay [30–32]. Numerous miRNAs have been confirmed to be involved in insecticide resistance by targeting detoxification enzymes or insecticide targets [33, 34]. LncRNAs are non-coding RNAs longer than 200 nucleotides that are structurally similar to messenger RNAs (mRNAs), but lack significant open reading frames [35, 36]. Generally, lncRNAs are classified into several categories, such as sense, antisense, intronic and intergenic, based on their position and direction of transcription in relation to protein-coding genes. Accumulated studies have shown that lncRNAs could regulate gene expression through a variety of mechanisms, including transcriptional regulation, post-transcriptional control and epigenetic modification [37]. In transcriptional level, lncRNA could span the promoter region of downstream protein encoding gene, prevent the binding of the transcriptional regulatory factors by acting as “decoy” or repress their activity by direct active-site occlusion or allosteric effects, thus inhibiting the expression of target genes; In contrast, lncRNAs could also promote transcriptional activity as enhancer RNAs or by binding to a protein with enhancer activity [38]. In post-transcriptional regulation, lncRNAs are implicated in the stability and translation of mRNAs, pre-mRNA splicing, protein activities, and act as precursors of miRNAs and siRNAs [39, 40]. In epigenetic level, lncRNAs regulate gene expression through DNA methylation or demethylation, RNA interference (RNAi), histone modification and chromosome remodeling [38, 39, 41]. In the last decades, plenty of studies on lncRNAs have been conducted in mammals. However, researches on the functions of lncRNAs in insecticide resistance, or even in agricultural insects, is still very limited. A lncRNA in intron 20 of the cadherin alleles was recently reported to regulate the transcription of cadherin 1 in the pink bollworm *Pectinophora gossypiella*, which also underlies its susceptibility to Cry1Ac [42]. Furthermore, an intergenic lncRNA, lincRNA_Tc13743.2, was reported to be involved in the upregulation of *GSTm02* by competing for miR-133-5p binding and thus mediated cyflumetofen resistance in *Tetranychus cinnabarinus* [43].

The present study was conducted aiming to investigate the role of *GSTu1* in chlorantraniliprole resistance, and the regulatory mechanisms of *GSTu1* by its antisense lncRNA (lnc-*GSTu1*-AS). Our results reveal a new ncRNA-mediated regulatory pathway that is involved in the control of detoxification enzymes in insecticide resistance and offer opportunities for further studies of the underlying mechanisms of insecticide resistance and even drug resistance.

## Results

### Cloning and expression pattern of *GSTu1* and lnc-*GSTu1*-AS between susceptible and chlorantraniliprole-resistant *P*. *xylostella* populations

**Fig 1:**
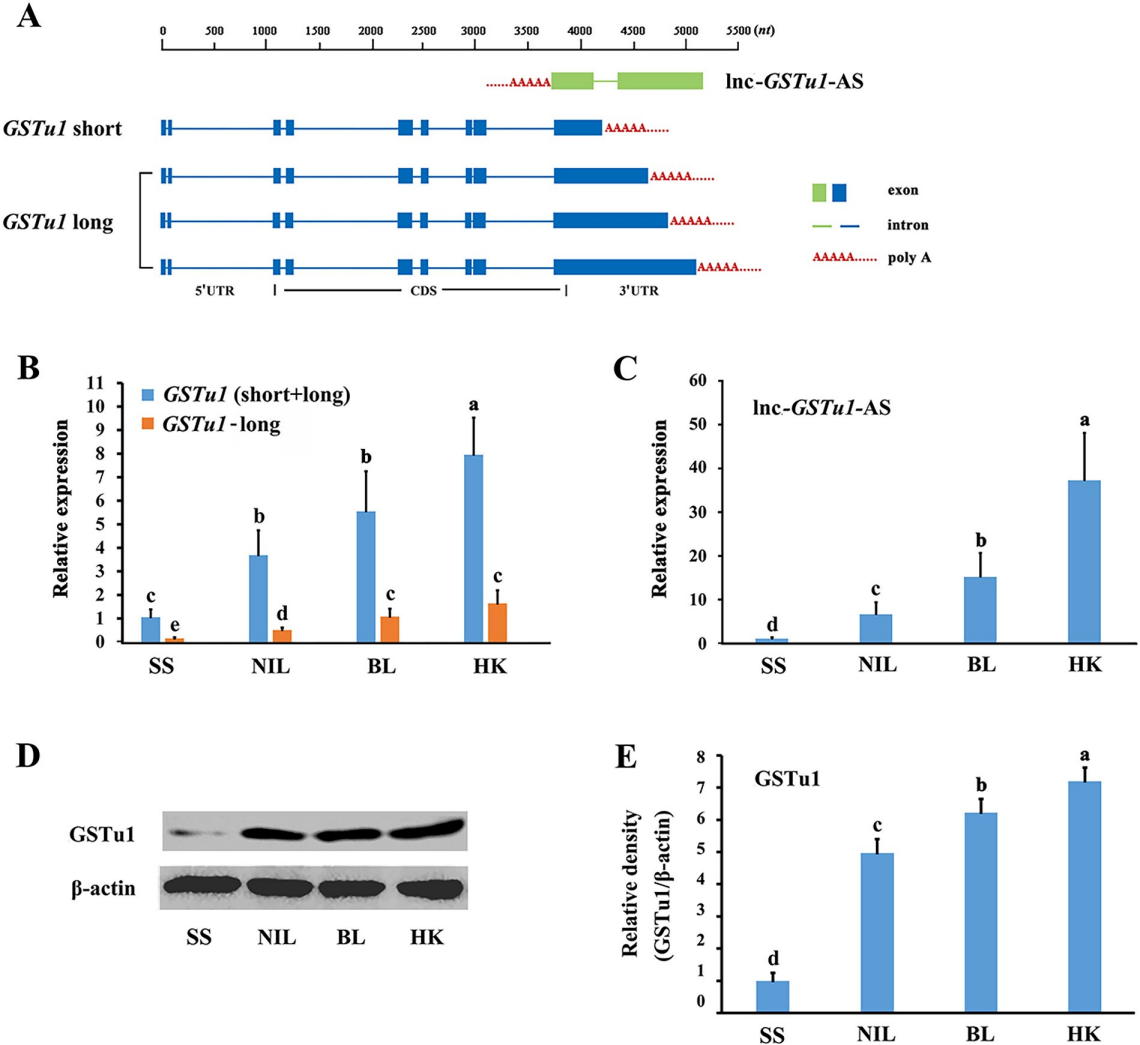
Structure and location of *GSTu1* and lnc-*GSTu1*-AS and their relative expression in susceptible and three chlorantraniliprole-resistant populations. A: The structure and location of full-length *GSTu1* and lnc-*GSTu1*-AS. Relative expression of GSTu1 in a susceptible population (SS) and three chlorantraniliprole-resistant populations (NIL, BL and HK) by qRT-PCR (B) and Western blotting (D, E). C: Relative expression of lnc-*GSTu1*-AS in a susceptible population (SS) and three chlorantraniliprole-resistant populations (NIL, BL and HK) by qRT-PCR. Different lowercase letters represent significant differences (one-way ANOVA followed by Tukey’s multiple comparison tests, *P* < 0.05).

Our previous transcriptome data showed that *GSTu1* and its antisense transcript lnc-*GSTu1*-AS were both upregulated in chlorantraniliprole-resistant *P*. *xylostella* populations [27]. The nucleotide sequences of *GSTu1* and lnc-*GSTu1*-AS were initially obtained from previous transcriptome sequencing [27] and then confirmed by reverse transcription PCR (RT-PCR) and rapid amplification of cDNA ends (RACE). Four *GSTu1* transcripts (GenBank accession number: MZ889082-MZ889085) with different 3’UTR lengths were obtained (Fig 1A).

Two field chlorantraniliprole-resistant *P*. *xylostella* populations were initially collected in vegetable fields in Boluo City, Guangdong Province (BL) and Haikou City, Hainan Province (HK) in 2016, respectively. A near-isogenic resistant population NIL was obtained from BL and a laboratory susceptible population SS. NIL, BL and HK showed 465-, 5057- and 6722-fold resistance to chlorantraniliprole compared with SS, respectively (S1 Table). Quantitative real-time polymerase chain reaction (qRT-PCR) results showed that the transcriptional levels of *GSTu1* in NIL, BL and HK larvae were 3.7-, 5.5- and 8.0-fold higher than those in SS larvae, respectively (Fig 1B). Moreover, the transcriptional level of the shortest transcript of *GSTu1* was much higher than that of the other three longer transcripts (Fig 1B). Based on their lengths, the shortest transcript was named “short”, and the three longer transcripts together were named “long” in this study (Fig 1A). The Western blot assay results showed that the expression of GSTu1 in NIL, BL and HK larvae was also higher than that in SS larvae (Fig 1D and 1E).

The nucleotide sequence of lnc-*GSTu1*-AS was identified by RT-PCR and RACE (GenBank accession number: MZ889086). Lnc-*GSTu1*-AS was transcribed from the opposite strand of *GSTu1* and overlapped almost the entire 3’UTR of *GSTu1* (Fig 1A) The qRT-PCR results showed that the transcriptional levels of lnc-*GSTu1*-AS in NIL, BL and HK larvae were 6.3-, 14.4- and 35.5-fold higher than those in SS larvae, respectively (Fig 1C).

The expressional level of both *GSTu1* and lnc-*GSTu1*-AS were gradually up-regulated with the increase of chlorantraniliprole resistance in NIL, BL and HK populations. It can be inferred that the role of *GSTu1* in chlorantraniliprole resistance is conserved and a similar regulatory relationship may exist between *GSTu1* and lnc-*GSTu1*-AS in these three resistant populations. Considering the NIL has very similar genetic background with SS, all subsequent *in vivo* verification experiments were performed using NIL.

### Sequence analysis of GSTu1

GSTs usually have two catalytically active sites: the prime GSH substrate binding site (G-site) and a secondary hydrophobic substrate binding site (H-site). The G-site within each GST class is formed by a group of highly conserved amino acid residues in the N-terminal domain of the protein. The H-site is formed by generally fewer conserved residues in the C-terminal domain [44]. Both G-sites and H-sites in the amino acid sequence of GSTu1 were analyzed with the NCBI CD-search program, and the results are shown in S1A Fig. Moreover, a phylogenetic tree was constructed based on the amino acid sequences of GSTu1 of 22 insect species from Lepidoptera, Diptera, Hymenoptera, Orthoptera and Coleoptera using the neighbor-joining method. The results showed that GSTu1 in Lepidoptera, including *P*. *xylostella*, was more conserved and clustered into a specific subclass (S1B Fig).

### Developmental and tissue expression patterns of *GSTu1* in *P*. *xylostella*

**Fig 2:**
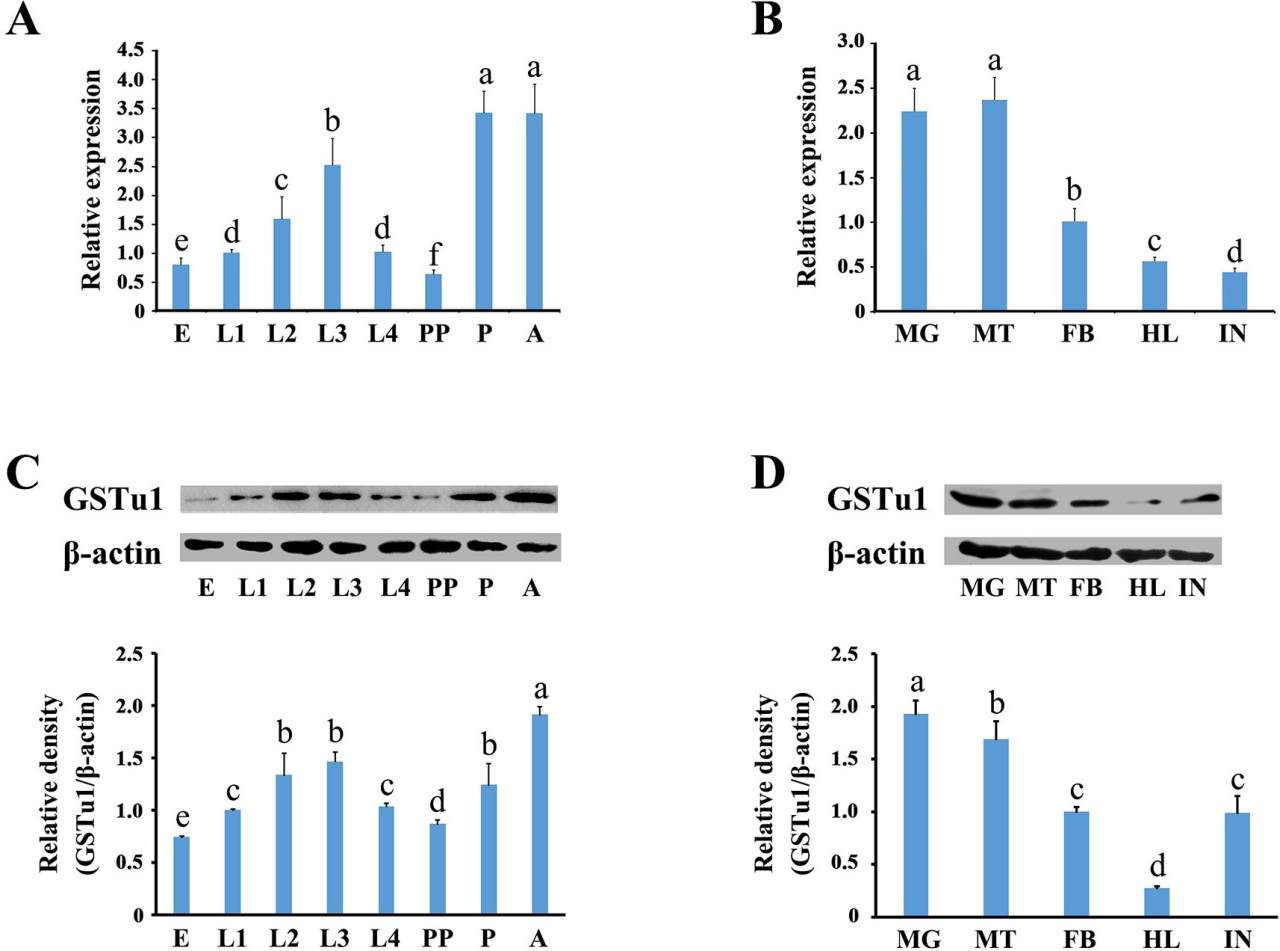
Expression of GSTu1 at different developmental stages and in different tissues. E: egg; L1-L4: the first- to fourth-instar larvae; PP: pre-pupae; P: pupae; A: adult. MG: midgut; MT: malpighian tubule; FB: fat body; HL: haemolymph; IN: integument. Different lowercase letters represent significant differences (one-way ANOVA followed by Tukey’s multiple comparison tests, *P* < 0.05).

The relative expression of *GSTu1* in different developmental stages and tissues was determined by qRT-PCR. The results showed that *GSTu1* was highly transcribed in adults, pupae and third instars, and abundant in the midgut and Malpighian tubules (Fig 2A and 2B). Similar results were also obtained by Western blot assay (Fig 2C and 2D).

### Functional analysis of GSTu1 in chlorantraniliprole resistance

**Fig 3:**
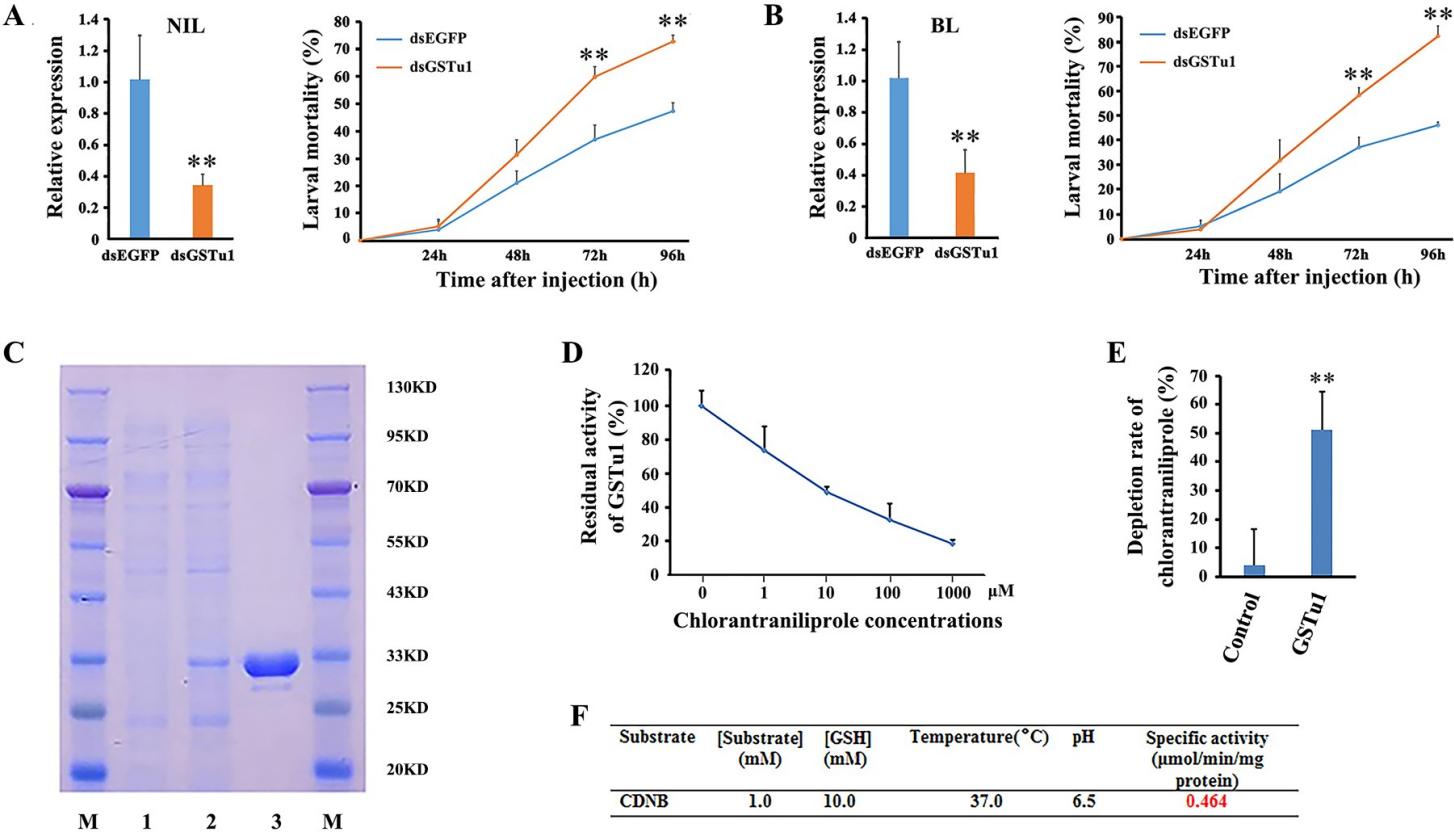
Functional analysis of GSTu1 in chlorantraniliprole resistance. RNAi-mediated knockdown of *GSTu1* reduced the resistance of *P*. *xylostella* to chlorantraniliprole in NIL (A) and BL(B). C: Expression and purification of GSTu1 in the *E*. *coli* system (M: protein marker; lane 1: negative control; lane 2: total soluble proteins; lane 3: purified proteins). D: Enzyme kinetics of GSTu1 with a series of ascending chlorantraniliprole (0–1000 μM) and fixed GSH. E: Depletion rate of chlorantraniliprole by recombinant GSTu1. F: The specific activity of purified GSTu1 toward CDNB. Asterisks indicate significant differences between the treatment and the corresponding control (Student’s *t*-test, * 0.01 < *P* < 0.05, **P < 0.01).

The function of *GSTu1* in chlorantraniliprole resistance was investigated by RNAi. The qRT-PCR results showed that the relative expression of *GSTu1* decreased by 70% and 62% in the NIL and BL populations, respectively, at 48 h postinjection of ds*GSTu1* compared with that of the control (injected with ds*EGFP*) (Fig 3A and 3B). Moreover, dsRNA-injected (ds*EGFP* and ds*GSTu1*) larvae from both NIL and BL were exposed to the LC_50_ of chlorantraniliprole. The mortality of the ds*GSTu1*-injected group was significantly increased by 17% and 22% in NIL and by 21% and 36% in BL at 72 h and 96 h postinjection, respectively (Fig 3A and 3B). These results indicated that RNAi-mediated knockdown of *GSTu1* increased the susceptibility of *P*. *xylostella* larvae to chlorantraniliprole.

Furthermore, recombinant GSTu1 was expressed as a soluble protein in *Escherichia coli*. The molecular mass of recombinant GSTu1 protein was approximately 30 KD, and more than 1 mg of recombinant protein was obtained after purification (Fig 3C). The specific activity of GSTu1 with CDNB as a substrate was 0.464 μmol/min/mg at pH 6.5 and 37°C (Fig 3F). The competitive assay revealed that chlorantraniliprole (1–1000 μM) was able to reduce the catalytic capability of recombinant GSTu1 by 26% to 82% (Fig 3D). This result indicated that the inhibition efficiency was dependent on the insecticide concentrations. The metabolism of chlorantraniliprole by recombinant GSTu1 was evaluated by High Performance Liquid Chromatography (HPLC). The results showed that approximately 47.4% of chlorantraniliprole was metabolized by recombinant GSTu1 compared with the control group (heat-inactivated GSTu1) (Fig 3E).

### Developmental and tissue expression patterns of lnc-*GSTu1*-AS in *P*. *xylostella*

**Fig 4:**
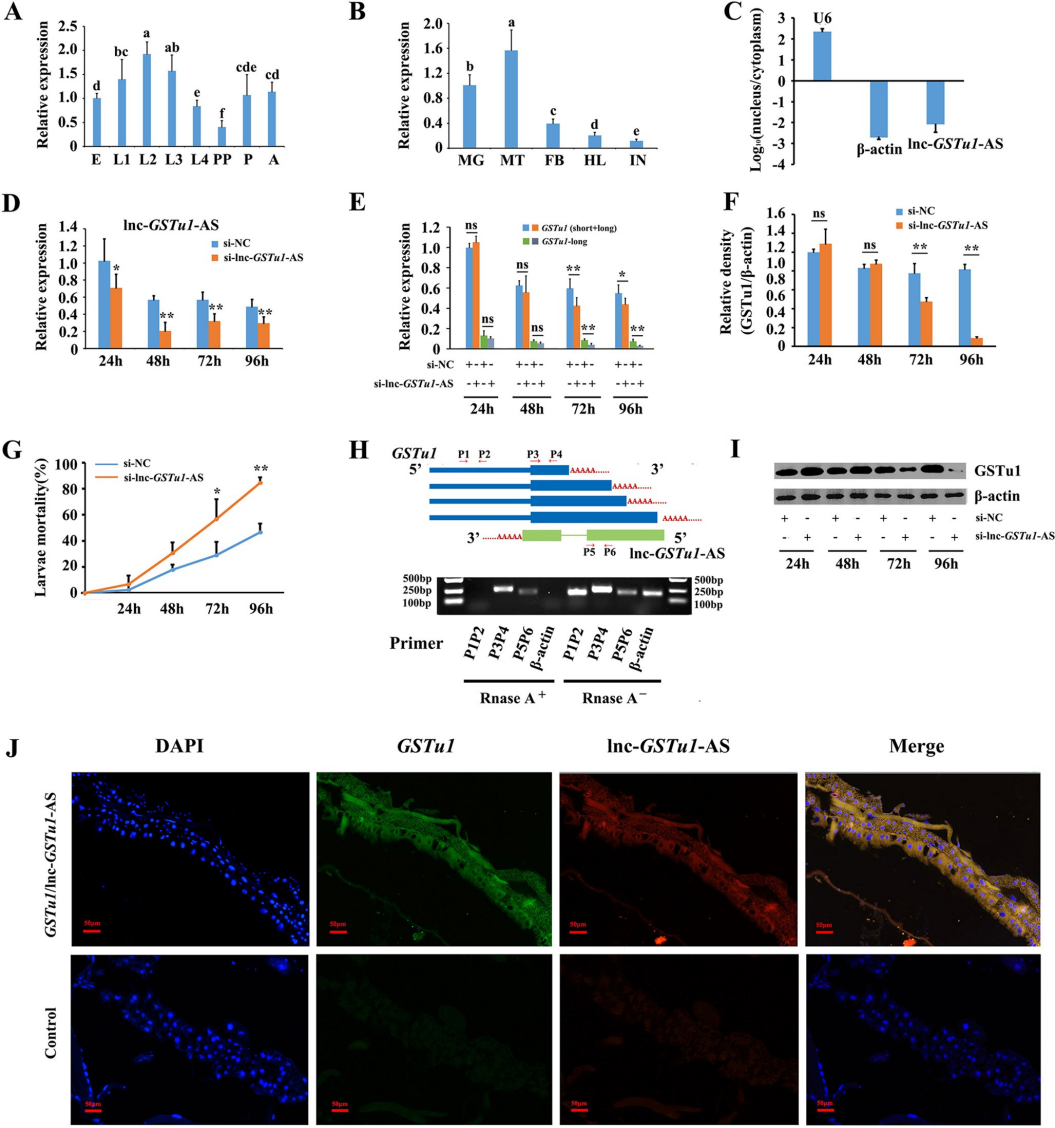
Lnc-*GSTu1*-AS is involved in the regulation of chlorantraniliprole resistance. Relative expression of lnc-*GSTu1*-AS at different developmental stages (A), in different tissues (B), in nuclear RNA and cytoplasmic RNA (C), and in control and lnc-*GSTu1*-AS knockdown groups (D) of *P*. *xylostella*. Relative expression of GSTu1 in the control and lnc-*GSTu1*-AS knockdown groups by qRT-PCR (E) and Western blot (F, I). G: Mortality of *P*. *xylostella* treated with the LC_50_ of chlorantraniliprole. H: Analysis of the binding relationship between lnc-*GSTu1*-AS and *GSTu1*-3′ UTR by ribonuclease protection assays (RPA). J: Co-localization of lnc-*GSTu1*-AS and *GSTu1* in the midgut of *P*. *xylostella* by fluorescence *in situ* hybridization (FISH) assay. Asterisks indicate significant differences between the treatment and the corresponding control (Student’s *t*-test, * 0.01 < *P* < 0.05, **P < 0.01). Different lowercase letters represent significant differences (one-way ANOVA followed by Tukey’s multiple comparison tests, *P* < 0.05).

The developmental and tissue-specific expression patterns of lnc-*GSTu1*-AS were also determined. qRT-PCR showed that lnc-*GSTu1*-AS was highly expressed in the first- to third-instar larval stage and abundant in the midgut and Malpighian tubules (Fig 4A and 4B). These results showed that *GSTu1* and lnc-*GSTu1*-AS had very similar expression patterns in different developmental stages and tissues of *P*. *xylostella*.

### Functional analysis of lnc-*GSTu1*-AS in resistance to chlorantraniliprole in *P*. *xylostella*

RNA fractionation of the nucleus and cytoplasm showed that lnc-*GSTu1*-AS was mainly abundant in the cytoplasm (Fig 4C), indicating its potential role in posttranscriptional regulation. The function of lnc*-GSTu1-*AS in chlorantraniliprole resistance was then investigated by RNAi in NIL. The qRT-PCR results showed that the relative expression of lnc*-GSTu1-*AS was suppressed by 31%, 64%, 44% and 40% at 24 h, 48 h, 72 h and 96 h after si-lnc*-GSTu1-*AS injection compared with that in the si-NC-injected control, respectively (Fig 4D). In response, the transcriptional level of *GSTu1* (short+long) decreased by 29% and 20%, and the transcriptional level of *GSTu1-*long decreased by 53% and 60%, at 72 h and 96 h post si-lnc*-GSTu1-*AS injection, respectively (Fig 4E). The Western blot assay results showed that the amount of GSTu1 also decreased significantly after silencing lnc*-GSTu1-*AS, especially 96 h post si-lnc*-GSTu1-*AS injection (Fig 4F and 4I). siRNA-injected larvae were exposed to the LC_50_ of chlorantraniliprole. The mortality in the si-lnc*-GSTu1-*AS injection group was increased by 28% and 38% at 72 h and 96 h (Fig 4G). These results indicated that RNAi-mediated knockdown of lnc*-GSTu1-*AS increased the susceptibility of *P*. *xylostella* larvae to chlorantraniliprole by reducing the expression of GSTu1.

The reverse complementary sequence between lnc*-GSTu1-*AS and *GSTu1* indicated that they might have a binding relationship. Therefore, the potential binding of lnc*-GSTu1-*AS directly to the 3’UTR of *GSTu1* was investigated. To detect the RNA duplex formed by lnc-*GSTu1*-AS and *GSTu1*, an RNase protection assay (RPA) was performed. The results showed that multiple sequence fragments (P3/P4, P5/P6) in the overlapping regions were protected from degradation, whereas the nonoverlapping region of lnc-*GSTu1*-AS and *GSTu1* (P1/P2) was almost completely degraded by RNase A (Fig 4H). Moreover, the colocalization signals of lnc-*GSTu1*-AS and *GSTu1* were also detected in the midguts of fourth instar NIL *P*. *xylostella* by the fluorescence *in situ* hybridization (FISH) assay, suggesting that these two transcripts formed a transient duplex (Fig 4J). Previous studies have shown that antisense lncRNAs promote the stability of sense transcripts by transient duplex formation and inhibition of miRNA- or RNA-binding protein (RBP)-induced sense mRNA decay [45]. Therefore, we assumed that lnc-*GSTu1*-AS might have a similar function in GSTu1 regulation.

### Identification of miRNAs targeting *GSTu1* in *P*. *xylostella*

RNA pull-down assays showed that no RBP interacted with *GSTu1* (S2 Fig). The results from two miRNA-target prediction software programs, miRanda and RNAhybrid, showed that 13 miRNAs could potentially act on the 3’UTR of *GSTu1*, and all the binding sites of these miRNAs were located in the overlap region of the sense-antisense transcripts (S3 Fig).

**Fig 5:**
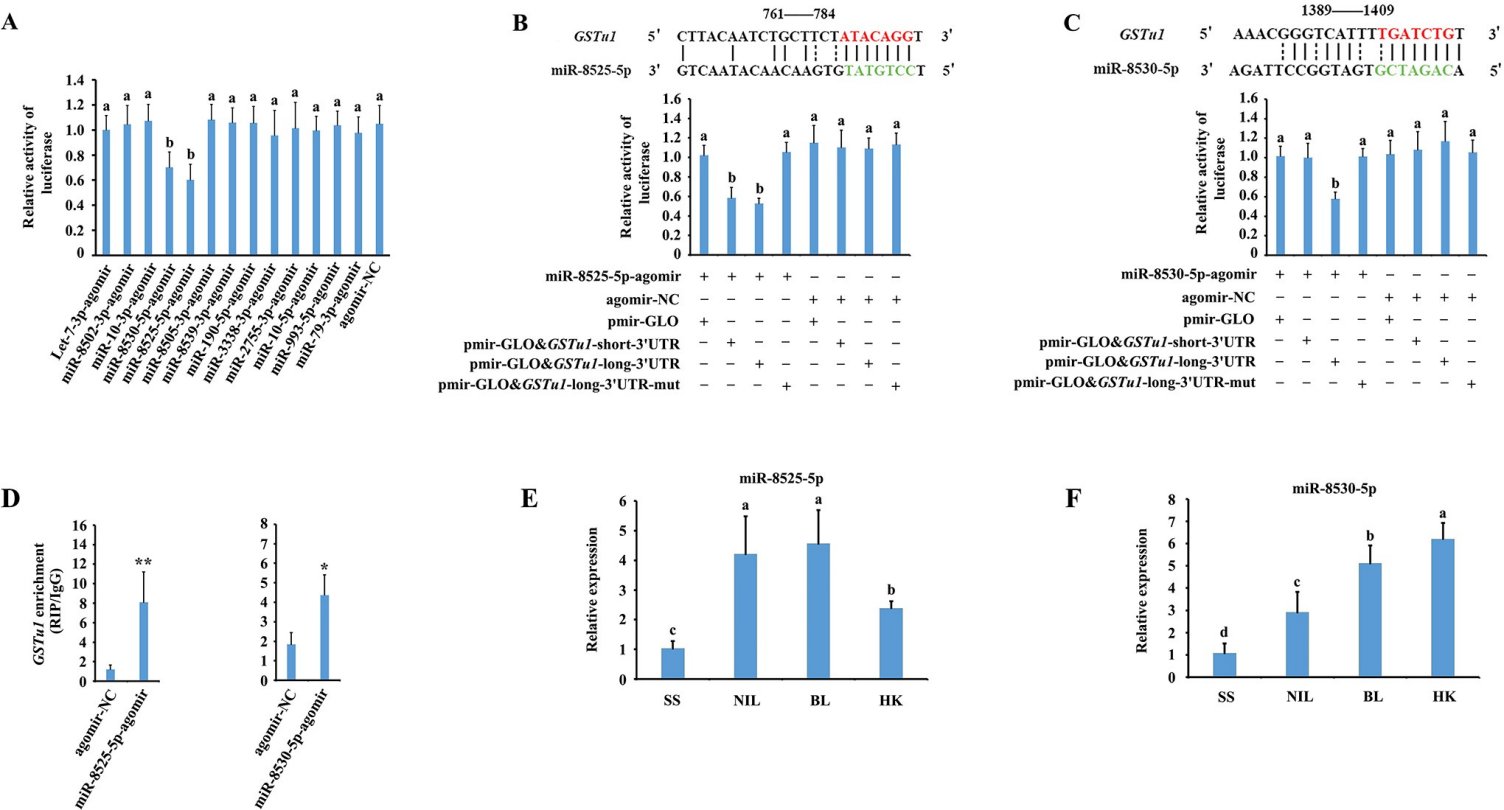
Identification of miRNAs targeting *GSTu1*. A: Validation of the interactions of 13 predicted miRNAs with *GSTu1* by dual-luciferase reporter assays. Dual-luciferase reporter assays through co-transfection of miR-8525-5p agomir (B) or miR-8530-5p agomir (C) with recombinant pmirGLO vectors containing either wild-type or mutated (mut) binding sites. D: Interactions between miR-8525-5p or miR-8530-5p and *GSTu1* determined by RNA-binding protein immunoprecipitation (RIP) *in vivo*. E: The relative expression of miR-8525-5p (E) and miR-8530-5p (F) in SS, NIL, BL and HK by RT-qPCR. Asterisks indicate significant differences between the treatment and the corresponding control (Student’s *t*-test, * 0.01 < *P* < 0.05, **P < 0.01). Different lowercase letters represent significant differences (one-way ANOVA followed by Tukey’s multiple comparison tests, *P* < 0.05).

To validate the binding of the 13 predicted miRNAs to *GSTu1* mRNA, the full-length 3’UTR sequence of the longest *GSTu1* transcript containing all the predicted miRNA-binding sites was initially cloned and inserted into a pmirGLO vector (pmirGLO & *GSTu1*-long-3’UTR). As a result, significant effects on reporter activity were only observed in miR-8525-5p- or miR-8530-5p agomir-transfected cells (Fig 5A). Next, the binding sites complementary to the ‘seed’ sequences of miR-8525-5p or miR-8530-5p were mutated (pmirGLO & *GSTu1*-long-3’UTR-mut). When the miR-8525-5p agomir was cotransfected with the pmirGLO & *GSTu1*-short-3’UTR or pmirGLO & *GSTu1*-long-3’UTR into HEK293T cells, the luciferase activity declined by 46% or 51.3%, respectively, compared with the mutated control (Fig 5B). When the miR-8530-5p agomir was cotransfected with the pmirGLO & *GSTu1*-long-3’UTR into HEK293T cells, the luciferase activity declined by 45.3% compared with the mutated control (Fig 5C). Furthermore, an RNA binding protein immunoprecipitation (RIP) assay was performed to determine whether *GSTu1* bound to miR-8525-5p- or miR-8530-5p-related RNA-induced silencing complexes (RISCs). The results showed that the levels of *GSTu1* significantly increased by 6.75-fold and 2.35-fold, respectively, when miR-8525-5p or miR-8530-5p was applied compared with the negative control (Fig 5D). These data indicated that *GSTu1* might interact directly with miR-8525-5p and miR-8530-5p. The binding site of miR-8525-5p was located at the overlapping 3’UTR sequence of all four *GSTu1* transcripts, while the binding site of miR-8530-5p was located at the overlapping 3’UTR sequence of the three longer *GSTu1* transcripts.

### The expression levels of miR-8525-5p and miR-8530-5p among SS, NIL, BL and HK

The expression levels of miR-8525-5p and miR-8530-5p were measured in SS, NIL, BL and HK by qRT-PCR. The results showed that the levels of miR-8525-5p in NIL, BL and HK larvae were 4.2-, 4.6- and 2.4-fold higher than those in SS larvae, respectively (Fig 5E), and the levels of miR-8530-5p in NIL, BL and HK larvae were 2.7-, 4.8- and 5.8-fold higher than those in SS larvae, respectively (Fig 5F).

### Upregulation and downregulation of miR-8525-5p or miR-8530-5p alone did not affect the expression of GSTu1

**Fig 6:**
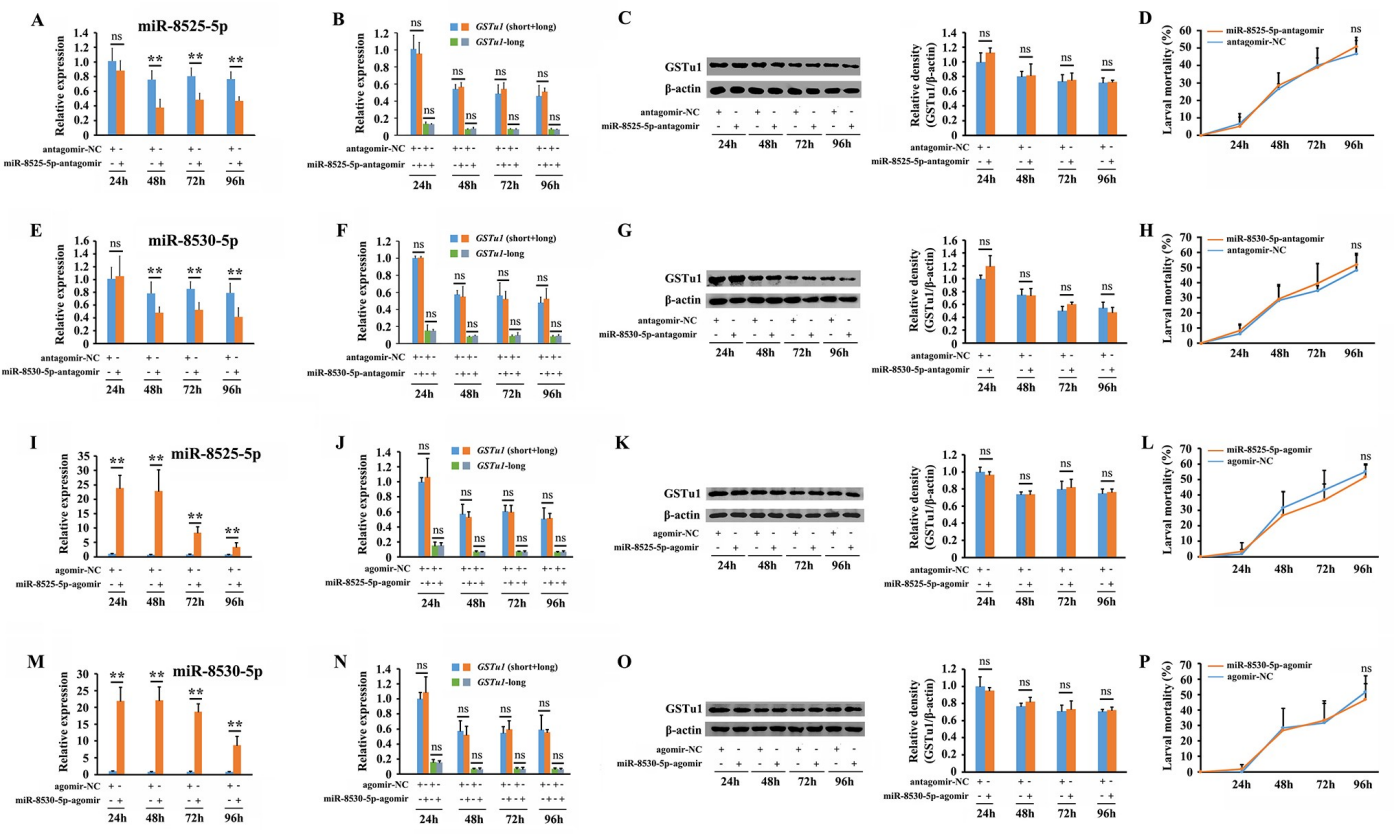
Effect of the upregulation and downregulation of miR-8525-5p or miR-8530-5p alone on the expression of GSTu1. Effects of miR-8525-5p antagomir/agomir treatment on the expression of miR-8525-5p (A, I) and GSTu1 (B, C, J, K) by qRT-PCR and Western blotting, as well as changes in susceptibility to chlorantraniliprole (D, L). Effects of miR-8530-5p antagomir/agomir treatment on the expression of miR-8530-5p (E, M) and GSTu1 (F, G, N, O) by qRT-PCR and Western blotting, as well as changes in susceptibility to chlorantraniliprole (H, P). Asterisks indicate significant differences between the treatment and the corresponding control (Student’s *t*-test, ns indicates no significant differences, * 0.01 < *P* < 0.05, **P < 0.01).

When the miR-8525-5p or miR-8530-5p antagomir was injected independently into the third instar of NIL, significant inhibition of miR-8525-5p or miR-8530-5p was observed compared with the antagomir-NC-injected control (Fig 6A and 6E). However, no significant alteration was detected at either the transcriptional or translational levels of *GSTu1* (Fig 6B, 6C, 6F and 6G), and no obvious difference was found between the mortalities of the antagomir injection group and the control (Fig 6D and 6H). Similar results were obtained when the miR-8525-5p or miR-8530-5p agomir was injected (Fig 6I–6P). These results indicated that upregulation and downregulation of miR-8525-5p or miR-8530-5p alone showed no effect on the expression of GSTu1.

### Lnc-*GSTu1*-AS enhanced the stability of *GSTu1* by preventing its degradation induced by miR-8525-5p in chlorantraniliprole-resistant *P*. *xylostella*

**Fig 7:**
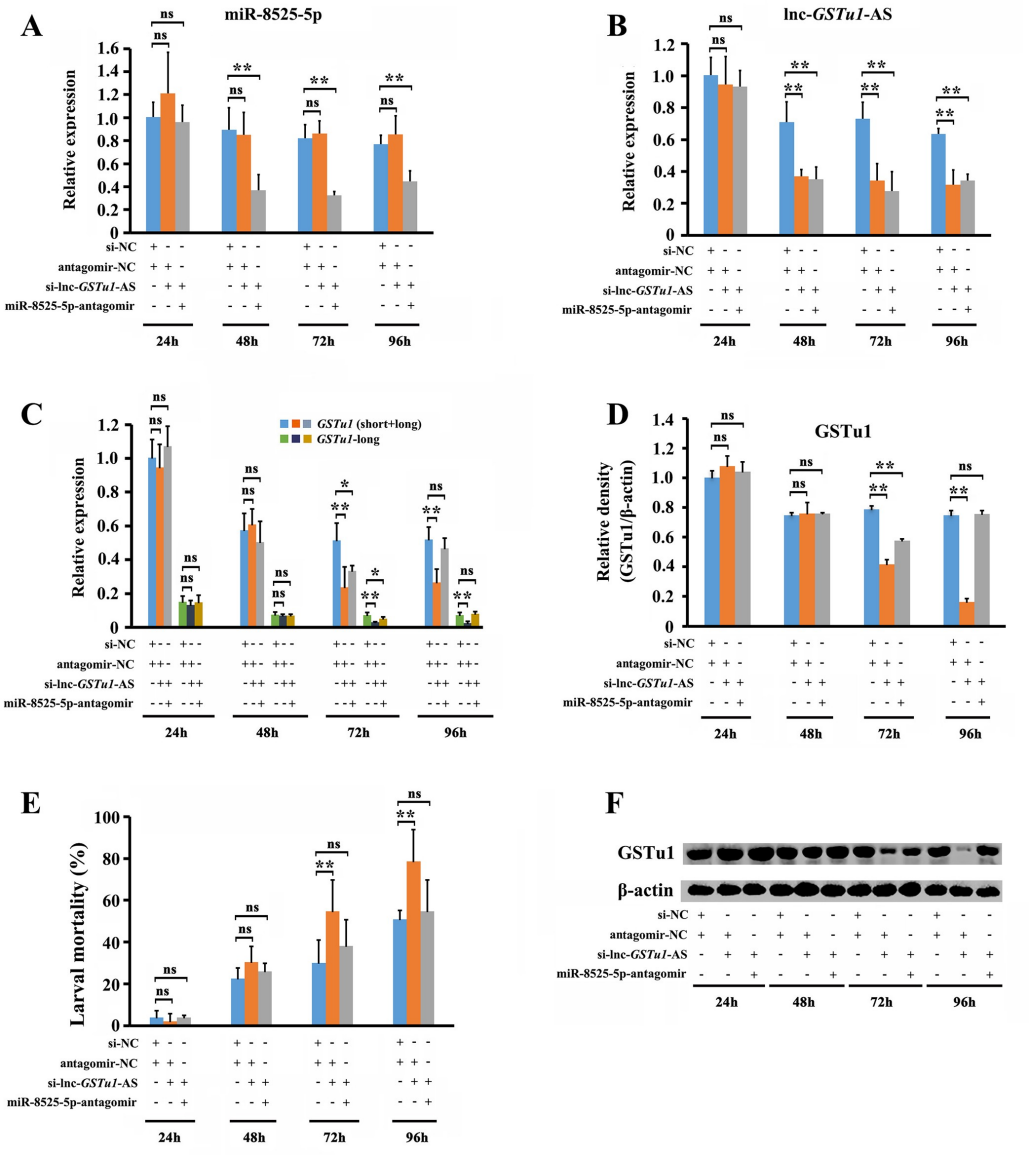
Lnc-*GSTu1*-AS maintains the stability of *GSTu1* through prevention of its degradation by masking the binding site of miR-8525-5p in chlorantraniliprole-resistant *P*. *xylostella*. Relative expression of miR-8525-5p (A) and lnc-*GSTu1*-AS (B) after inhibition of miR-8525-5p and knockdown of lnc-*GSTu1*-AS by qRT-PCR. Relative expression of GSTu1 after inhibition of miR-8525-5p combined with knockdown of lnc-*GSTu1*-AS by qRT-PCR (C) and Western blot assay (D, F). E: Mortality of *P*. *xylostella* treated with the LC_50_ of chlorantraniliprole. Asterisks indicate significant differences between the treatment and the corresponding control (Student’s *t*-test, ns indicates no significant differences, * 0.01 < *P* < 0.05, **P < 0.01).

To further investigate whether the effect of lnc-*GSTu1*-AS on the stability of *GSTu1* was associated with miRNAs, we first inhibited miR-8525-5p or miR-8530-5p by injecting antagomirs (antagomir-NC was used as a control) and then (12 h later) knocked down the expression of lnc-*GSTu1*-AS by injecting si-lnc-*GSTu1*-AS (si-NC was used as a control) into 3rd instar NIL larvae. The relative expression of miR-8525-5p was significantly decreased by 58.8%, 60.6% and 42% at 48 h, 72 h and 96 h postinjection of miR-8525-5p-antagomir/si-lnc-*GSTu1*-AS (Fig 7A). The relative expression of lnc-*GSTu1*-AS was significantly downregulated in both the miR-8525-5p-antagomir/si-lnc-*GSTu1*-AS (by 50.5%, 62.1% and 46.1% at 48 h, 72 h and 96 h postinjection) and antagomir-NC/si-lnc-*GSTu1*-AS (by 47.8.5%, 53.3% and 50.1% at 48 h, 72 h and 96 h postinjection) injection groups (Fig 7B).

As an influence, the transcriptional level of *GSTu1* (short+long) in the group injected with antagomir-NC/si-lnc-*GSTu1*-AS decreased by 54.0% and 49.8%, and the transcriptional level of *GSTu1*-long decreased by 54.0% and 49.8%, at 72 h and 96 h, respectively, after siRNA injection compared with that in the control (antagomir-NC/si-NC injection group) (Fig 7C). Similar results were also obtained at the translational level of GSTu1 by Western blot (Fig 7D and 7F). The mortality of larvae exposed to the LC_50_ of chlorantraniliprole was 78.7%, which was significantly higher than that in the antagomir-NC/si-NC group (50.9%) (Fig 7E).

The transcriptional level of *GSTu1* (short+long) in the group injected with miR-8525-5p-antagomir/si-lnc-*GSTu1*-AS decreased by 35.1%, and the transcriptional level of *GSTu1*-long decreased by 30.3% (Fig 7C), only at 72 h after siRNA injection, while no significant difference was found at 96 h after siRNA injection compared with the control (antagomir-NC/si-NC-injected group). Similar results were also obtained at the translational level of GSTu1 by Western blot (Fig 7D and 7F). Furthermore, the mortality of the larvae exposed to the LC_50_ of chlorantraniliprole was 54.6%, which was significantly lower than that in the antagomir-NC/si-lnc-*GSTu1*-AS-injected group (78.7%) (Fig 7E).

In summary, these results suggested that knockdown of lnc-*GSTu1*-AS alone increased the susceptibility of larvae to chlorantraniliprole, while suppression of both lnc-*GSTu1*-AS and miR-8525-5p did not. It means that lnc-*GSTu1*-AS maintained the stability of *GSTu1* by preventing its degradation induced by miR-8525-5p, and thus mediated chlorantraniliprole resistance in *P*. *xylostella*.

### miR-8530-5p is not involved in the regulation of GSTu1 mediated by lnc-*GSTu1*-AS

**Fig 8:**
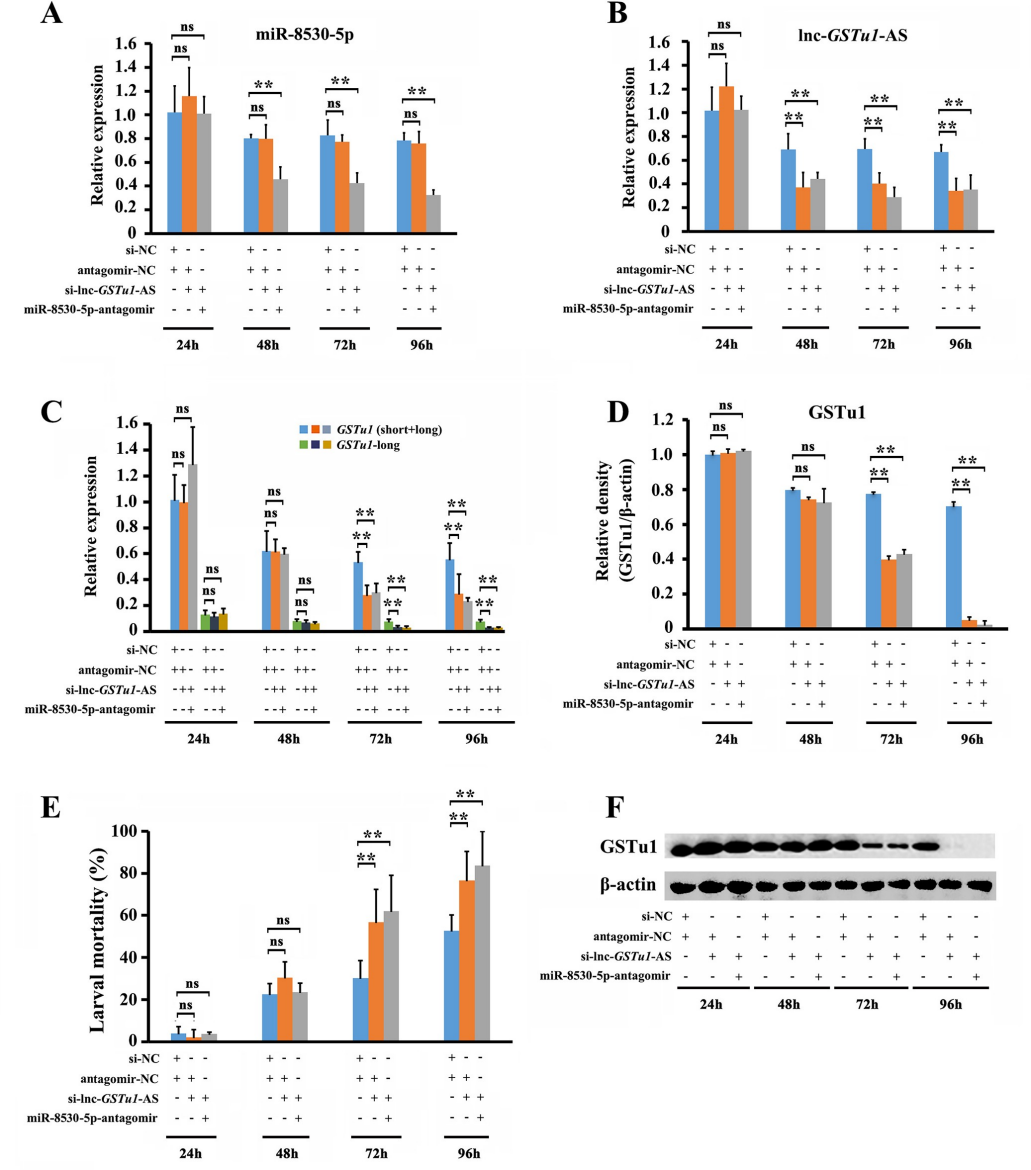
miR-8530-5p is not involved in the regulation of *GSTu1* by lnc-*GSTu1*-AS. Relative expression of miR-8530-5p (A) and lnc-*GSTu1*-AS (B) after inhibition of miR-8530-5p and knockdown of lnc-*GSTu1*-AS. C: Relative expression of GSTu1 after inhibition of miR-8530-5p and knockdown of lnc-*GSTu1*-AS by qRT-PCR (C) and Western blot assay (D, F). E: Mortality of *P*. *xylostella* treated with the LC_50_ of chlorantraniliprole. Asterisks indicate significant differences between the treatment and the corresponding control (Student’s *t*-test, ns indicates no significant differences, * 0.01 < *P* < 0.05, **P < 0.01).

The abundance of miR-8530-5p significantly decreased by 43%, 48.6% and 58.8% at 48 h, 72 h and 96 h, respectively, after injection of miR-8530-5p-antagomir/si-lnc-*GSTu1*-AS (Fig 8A), and the expression of lnc-*GSTu1*-AS was significantly downregulated in both groups injected with miR-8530-5p-antagomir/si-lnc-*GSTu1*-AS (by 35.6%, 58.6% and 47.4% at 48 h, 72 h and 96 h postinjection) and antagomir-NC/si-lnc-*GSTu1*-AS (by 46.1%, 41.9% and 49.2% at 48 h, 72 h and 96 h postinjection) (Fig 8B).

In response, the transcriptional level of *GSTu1* (short+long) decreased by 47.8% and 47.9% in the antagomir-NC/si-lnc-*GSTu1*-AS-injected group and 43.8% and 58.4% in the miR-8530-5p-antagomir/si-lnc-*GSTu1*-AS-injected group (Fig 8C), respectively, at 72 h and 96 h post siRNA injection compared with that in the control (antagomir-NC/si-NC-injected group). Meanwhile, the transcriptional level of *GSTu1*-long exhibited the similar change (Fig 8C). Similar results were also obtained for the translational levels of GSTu1 by Western blot assay (Fig 8D and 8F). Furthermore, the 96-h mortality of the larvae exposed to the LC_50_ of chlorantraniliprole was 76.6% in the antagomir-NC/si-lnc-*GSTu1*-AS-injected group and 83.7% in the miR-8530-5p-antagomir/si-lnc-*GSTu1*-AS-injected group, both of which were significantly higher than that in the antagomir-NC/si-NC-injected group (52.7%) (Fig 8E).

In summary, these results suggested that either knockdown of lnc-*GSTu1*-AS alone or suppression of both lnc-*GSTu1*-AS and miR-8530-5p could increase the susceptibility of larvae to chlorantraniliprole. Thus, miR-8530-5p was not involved in the regulation of GSTu1 mediated by lnc-*GSTu1*-AS, potentially because miR-8530-5p could only act on the three longer transcripts (*GSTu1*-long), which had a much lower abundance among all four *GSTu1* transcripts and might not contribute to chlorantraniliprole resistance.

## Discussion

GST is an important detoxification enzyme that can catalyze glutathione (GSH) conjugation to chemical substances to increase their solubility and facilitate their excretion from the cell in animals [44]. Previous studies have shown that GSTs contribute to insecticide resistance not only by direct metabolism or sequestration of chemicals but also indirectly by providing protection against oxidative stress induced by insecticide exposure [44]. In the present research, GSTu1 in *P*. *xylostella* was confirmed to be involved in chlorantraniliprole resistance by RNAi and *in vitro* metabolism experiments. RNAi-mediated knockdown of *GSTu1* in two chlorantraniliprole-resistant populations reduced the resistance of *P*. *xylostella* to chlorantraniliprole. Moreover, the activity of recombinant GSTu1 protein was inhibited by chlorantraniliprole, which suggested that the enzyme might contribute to insecticide resistance in *P*. *xylostella* [46]. The interaction between GSTu1 and chlorantraniliprole was further investigated by HPLC analysis. Recombinant GSTu1 exhibited significant decomposition ability against chlorantraniliprole. Although GSTs have been reported to be involved in the metabolism of organochloride [47, 48], organophosphorus [49] and pyrethroid [50] insecticides in insects, as well as the metabolism of cyflumetofen in spider mites [51], this is the first report of the involvement of GSTs in the direct metabolism of diamide insecticides.

Antisense lncRNAs are transcribed from the opposite DNA strand to their paired sense protein coding genes and can enhance mRNA stability by blocking miRNA activity. For example, lncRNA BACE1-AS, which is partially antisense to BACE1 (a gene encoding the β-site amyloid precursor protein cleaving enzyme 1), has been identified to be markedly upregulated in the brains of Alzheimer’s disease patients and promotes the stability of its sense partner (BACE1) [45]. Further experiments revealed that lncRNA BACE1-AS regulates the expression of BACE1 by preventing miRNA-induced mRNA decay and translational repression [52]. Similarly, polypyrimidine tract-binding protein 1 (PTB1) antisense RNA (PTB1-AS) can also modulate its sense mRNA stability by masking miRNA binding sites. Specifically, PTB1-AS binds to the 3’UTR of PTBP1 and prevents miR-9 (a neural-specific miRNA that is known to target the 3’UTR of PTBP1) binding for degradation [53].

Here, we identified an antisense lncRNA, lnc-*GSTu1*-AS, which is partially antisense to *GSTu1* in *P*. *xylostella*. The qRT-PCR results showed that lnc-*GSTu1*-AS and *GSTu1* were both upregulated in chlorantraniliprole-resistant populations. Furthermore, we discovered that lnc-*GSTu1*-AS colocalized with *GSTu1* mRNA, which suggested that lnc-*GSTu1*-AS might be a *cis*-regulatory factor of *GSTu1*. Current research efforts have focused on the regulatory mechanisms of insecticide resistance-associated genes [54]. A series of *trans*- and *cis*-regulatory elements have been identified to mediate detoxification gene expression in insecticide-resistant insects, especially transcription factors [54, 55] and miRNAs [33, 34]. Methylation of mRNA was recently confirmed to be responsible for the regulation of the expression of the cytochrome P450 gene (*CYP4C64*), which in turn is responsible for insecticide resistance in the global whitefly pest *Bemisia tabaci* [56]. However, very few studies have strongly revealed the functions of lncRNAs in the regulation of resistance-associated genes. RNAi-based knockdown of lnc-*GSTu1*-AS reduced the expression of *GSTu1*, especially on the protein level. One important role of miRNA is to inhibit the translation of the target genes. Thus, we inferred that lnc-*GSTu1*-AS might play a crucial role in maintaining the stability of *GSTu1* by preventing miRNA-induced translational repression. Then two miRNAs, miR-8525-5p and miR-8530-5p, were identified to be targeted to *GSTu1* by the dual-luciferase reporter assay and RIP. However, injection of miR-8525-5p or miR-8530-5p agomir/antagomir alone did not affect the expression level of *GSTu1* or the sensitivity of *P*. *xylostella* to chlorantraniliprole. We speculated that the antisense lncRNA might have blocked the binding of miRNAs to *GSTu1*. Moreover, the expression levels of *GSTu1* and lnc-*GSTu1*-AS might have been kept in a dynamic balance after long-term exposure to chlorantraniliprole; therefore, changes in miRNA abundance alone could not affect the expression of *GSTu1*.

**Fig 9:**
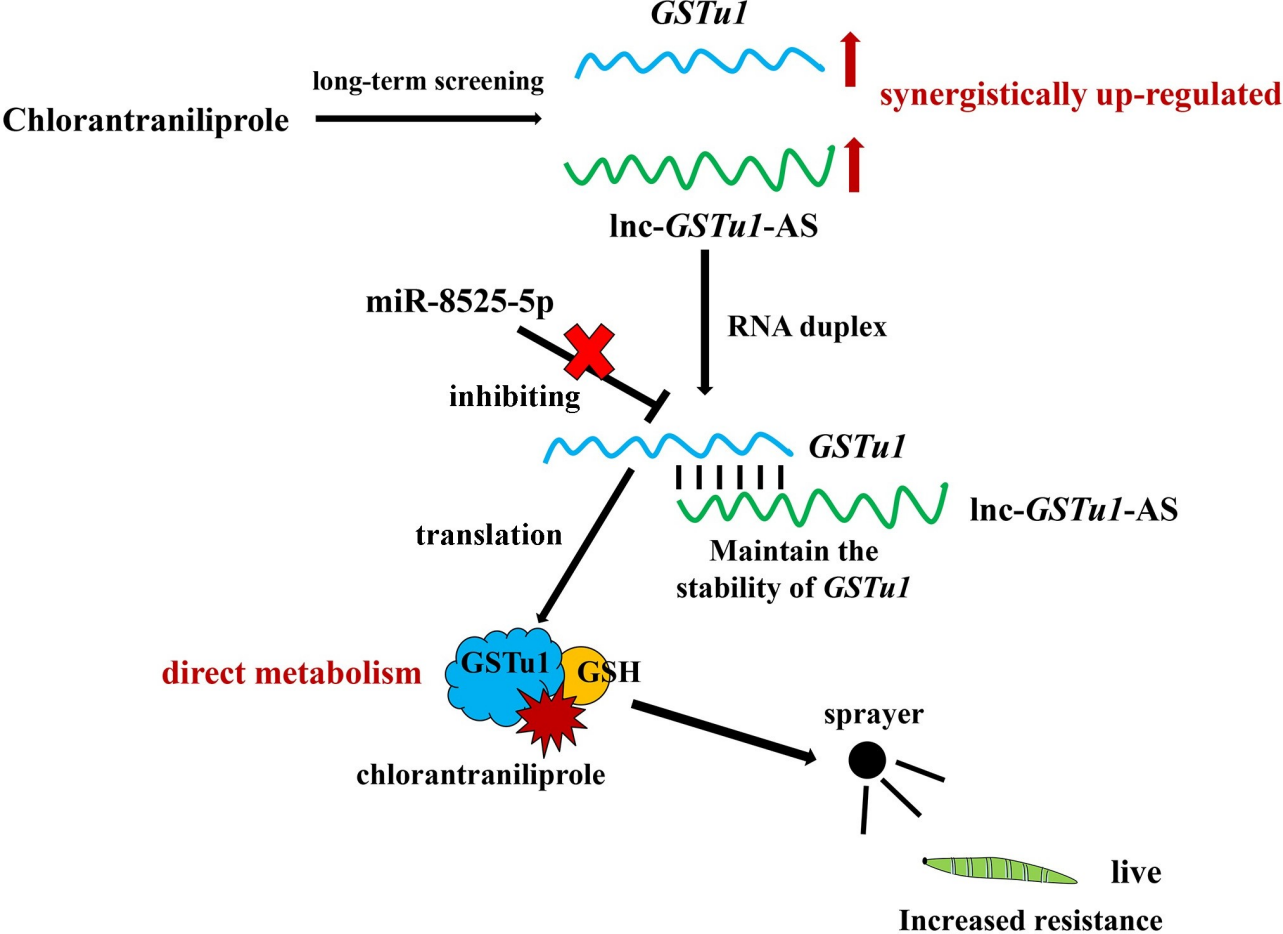
A schematic illustration of the proposed model depicting the role of lnc-*GSTu1*-AS in regulating *GSTu1* mRNA stability.

Inhibition of miR-8525-5p, along with knockdown of lnc-*GSTu1*-AS, effectively prevented the decrease in GSTu1 expression, thereby maintaining the resistance of *P*. *xylostella* to chlorantraniliprole. In detail, lnc-*GSTu1*-AS bound to the 3’UTR of *GSTu1* and thus prevented its degradation caused by the binding of miR-8525-5p (Fig 9). Although miR-8530-5p could also bind to the 3’UTR of *GSTu1*, it only acted on the three longer transcripts of *GSTu1*. The abundance of the three longer transcripts was too low and could not have a significant impact on chlorantraniliprole resistance. Although it was very difficult to verify the function of lnc-*GSTu1*-AS through *in vivo* overexpression and bioassays, considering that the injection of their respective agomir or antagomir caused an up- or a downregulation of miR-8525-5p or miR-8530-5p alone and showed no effect on the expression of GSTu1, together with the high expression of miR-8525-5p and miR-8530-5p in chlorantraniliprole-resistant populations, we inferred that the abundance of lnc-*GSTu1*-AS might have provided sufficient protection for *GSTu1* in the resistant population and thus maintained resistance to chlorantraniliprole.

In conclusion, our results revealed that lnc-*GSTu1*-AS stabilized the expression of *GSTu1* by masking the binding site of miR-8525-5p at the 3’UTR of *GSTu1* and thus increased the metabolism of chlorantraniliprole by GSTu1 (Fig 9). To our knowledge, this is a novel mechanism that regulates GST-mediated metabolic resistance and the first report of the promotion of complementary mRNA stability by its antisense lncRNA in insects. The results provide further insights into the mechanisms underlying metabolic resistance, as well as new ideas for further and more in-depth research on the mechanisms underlying insecticide resistance.

## Materials and methods

### Insects

The susceptible population (SS) of *P*. *xylostella* was initially purchased from the Pilot-Scale Base of Bio-Pesticides, Institute of Zoology, Chinese Academy of Sciences in 2014 and has been maintained in our laboratory at China Agricultural University for seven years without exposure to any insecticide. The field-resistant *P*. *xylostella* population BL was collected in vegetable fields in Boluo, Guangdong Province, in 2016 and then selected with chlorantraniliprole intermittently to maintain resistance. NIL is a near-isogenic resistant population that was obtained from SS and BL as previously [57]. The field-resistant population HK was collected from vegetable fields in Haikou, Hainan Province in 2016 and then constantly selected with chlorantraniliprole in our laboratory. NIL, BL and HK showed 465-, 5057- and 6722-fold resistance to chlorantraniliprole compared with SS, respectively (S1 Table). All stages of *P*. *xylostella* were maintained at 27 ± 1°C, with a photoperiod of 16 h light:8 h dark on radish seedlings (*Raphanus sativus* L.). Adults were provided with 10% (w/v) honey solution and allowed to lay eggs on radish seedlings.

### Bioassay

The leaf-dipping method [58] was used to determine the toxicity of chlorantraniliprole. Cabbage leaves were dipped in the required concentrations of chlorantraniliprole solution for 15 s and then shade-dried for 20 min. A 0.1% (v/v) Triton X-100 water solution was used as a control. Approximately 20 third instars were transferred onto each leaf, and three replications were used for each concentration. The mortality was observed at 96 h posttreatment. LC_50_ values were calculated using POLO-Plus 2.0 software (LeOra Software Inc., Berkeley, CA).

### Total RNA isolation and cDNA synthesis

Total RNA was extracted using TRIzol reagent (Invitrogen, Carlsbad, CA, USA) following the manufacturer’s protocol. Samples were collected from each *P*. *xylostella* population (SS, NIL, BL, HK). Developmental-specific samples were collected from eggs, first- to fourth-instar adults, prepupae, pupae and first-day adults (equal numbers of females and males). Tissue samples were collected from the head, midgut, integument, fat body and Malpighian tubules of the fourth instar. First-strand cDNA was prepared from total RNA by reverse transcription using a PrimeScript RT reagent kit with gDNA Eraser (TaKaRa, Dalian, China) following the manufacturer’s recommendations.

### Cloning and sequence analysis of *GSTu1* and lnc-*GSTu1*-AS in *P*. *xylostella*

The nucleotide sequence of *GSTu1* and its antisense transcript (named lncRNA-*GSTu1*-AS) were initially obtained from our previous transcriptome data [27]. RT-PCR was used to validate the sequences of these two transcripts. The program was as follows: 94°C for 3 min; followed by 35 cycles at 94°C for 30 s, 60°C for 30 s and 72°C for 1 min; and a final extension at 72°C for 10 min. The RACE cDNA Amplification kit (Clontech, Mountain View, CA, USA) was used to clone the full-length sequences of *GSTu1* and lnc-*GSTu1*-AS. Gene-specific primers for 5′-RACE and 3′-RACE were designed based on known fragments of these two transcripts. The first-round PCRs were performed with the gene-specific primers and universal primer mix (UPM) supplied in the kit, and the program was as follows: 94°C for 3 min; 5 cycles at 94°C for 30 s and 72°C for 3 min, followed by 25 cycles at 94°C for 30 s, 68°C for 30 s, and 72°C for 3 min; and a final extension at 72°C for 10 min. For the second-round PCR, the first-round PCR products were diluted 50–100 times and then used as templates. The second-round PCR program was as follows: 94°C for 3 min; followed by 20 cycles at 94°C for 30 s, 68°C for 30 s and 72°C for 3 min; and a final extension at 72°C for 10 min.

The putative GSTu1 amino acid sequences of *P*. *xylostella* and other related insects were aligned using Clustal X2.0, and then a phylogenetic tree was constructed using the neighbor-joining method with MEGA6 software based on a bootstrap analysis with 1000 replications.

### Validation of gene expression by qRT-PCR

qRT-PCR was performed to experimentally validate the relative expression of the *GSTu1* and lnc-*GSTu1*-AS transcripts. *β-actin* and *EF1a* were used as internal controls. qRT-PCR was conducted in an ABI 7500 Real-Time PCR System (Applied Biosystems, Foster City, CA, USA). The SYBR Premix Ex Taq kit (TaKaRa) was used with a standard PCR protocol, and the PCR program was as follows: 95°C for 30 s, followed by 40 cycles at 95°C for 5 s and 60°C for 34 s. Melting curves were analyzed by heating the sample to 95°C for 15 s and then cooling them to 60°C for 15 s, and melting curves were then obtained by autoincrements of 1°C/15 s for each cycle until 95°C was reached. The relative expression of each transcript was calculated using the 2^–ΔΔCt^ method [59].

### RNA interference and miRNA injection

The dsRNA of *GSTu1* was prepared *in vitro* using the MEGAscript RNAi kit (Ambion, Foster City, CA, USA). Gene-specific primers containing a T7 polymerase promoter sequence were designed on the E-RNAi website (a tool for the design and evaluation of RNAi reagents for a variety of species, http://www.dkfz.de/signaling/e-rnai3/). The dsRNA of enhanced green fluorescent protein (EGFP) was used as a control. All of the synthesized dsRNAs were dissolved in nuclease-free water and then quantified using a NanoDrop 2000 (Thermo Scientific, Wilmington, DE, USA). A siRNA targeting lnc-*GSTu1*-AS (si-lnc-*GSTu1*-AS) was designed in the nonoverlapping region between *GSTu1* and lnc-*GSTu1*-AS and synthesized by Shanghai Genepharm Co., Ltd. (Shanghai, China). A negative control-siRNA (NC-siRNA) was used as a control. GSTu1 dsRNA and si-lnc-*GSTu1*-AS were injected into early third instars (400 ng/individual) using a microinjector (Nanoliter 2000 Injector; WPI Inc., Sarasota, FL, USA). miRNA mimics (agomir), inhibitors (antagomir) and their respective negative controls (NC agomir, NC antagomir) were synthesized by Shanghai Genepharm Co., Ltd. (Shanghai, China) (S2 Table). An aliquot of 138 nL of 50 μM miRNA agomir or antagomir was injected into each third instar larva, and the control was injected with the same amount of NC agomir or antagomir. A sample of the injected insects was collected for later detection of gene expression, and the remaining insects were used for parallel bioassays to determine the sensitivity of the injected larvae to chlorantraniliprole. The leaf-dipping method described above was used to treat the injected larvae with an LC_50_ of chlorantraniliprole. The mortalities were recorded at 96 h posttreatment. The experiments were performed with three replicates. A schematic representation of all injection experiments is shown in S4 Fig.

### Expression and purification of GSTu1

Sequencing-verified *GSTu1* was digested with BamHI and EcoRI and then inserted into the expression vector pET-28a (+), followed by transformation into *E*. *coli* BL21 (DE3) cells for expression. Expression of recombinant GSTu1 protein was induced in 500 mL Luria–Bertani (LB) liquid medium with 0.2 mM isopropyl β-d-thiogalactoside (IPTG), which was continued for 12 h with shaking at 150 rpm at 30°C. Induced bacteria were harvested by centrifugation at 12 000 g and 4°C and were resuspended in 10 mL of 10 mM Tris-HCl buffer (pH 8.0). After sonication for 5 min, the collected bacteria were centrifuged for 10 min at 12 000 g and 4°C. The soluble fraction of recombinant GSTu1 was identified by SDS-PAGE. Recombinant protein was bound by Ni-NTA His Bind Resin (Abiotech, Jinan, China) and eluted using an ascending series of imidazoles (20, 50, 150 and 250 mM). The purified protein was dialyzed using 10 mM Tris-HCl buffer with 150 mM NaCl and 50% glycerol. A BCA (Bicin-choninic Acid) Protein Assay Kit (Cwbiotech, Beijing, China) was used to determine the concentration of recombinant GSTu1.

### *In vitro* enzyme kinetics and metabolism assays

The specific activities of recombinant GSTu1 were detected using the method described by Zhang et al. [60]. The total reaction volume of 200 μL included 4 μL of recombinant GSTu1 protein, 196 μL of 50 mM phosphate-buffered saline (PBS, pH 6.5) containing 1 mM CDNB, and 10 mM glutathione (GSH) to monitor the OD340 for 2 min in an Absorbance Microplate Reader (SpectraMax, Molecular Devices, Sunnyvale, California, USA) at 37°C. The inhibition of chlorantraniliprole against recombinant GSTu1 was also measured. The total reaction volume of 200 μL included 4 μL of recombinant GSTu1 protein, 196 μL of 50 mM PBS (pH 6.5) containing 1 mM CDNB, 10 mM GSH, and different concentrations of chlorantraniliprole (1, 10, 100, 1000 μM). The enzyme activity was determined as described above. The same volume of 50 mM PBS (pH 6.5) and heat-inactivated GSTu1 was used as a negative control, and each test was repeated three times.

To determine the capacity of GSTu1 to metabolize chlorantraniliprole, *in vitro* assays were performed adapted from Badawy [61]. A total of 500 μL of the reaction system containing 0.1 M PBS (pH 7.2), 2.5 mM GSH, 0.45 mM chlorantraniliprole, and 20 μg of recombinant GSTu1 protein was incubated at 30°C for 4 h with shaking at 200 rpm. Subsequently, 500 μL of methanol (high-performance liquid chromatography, HPLC grade) was added to stop the reaction. The samples were then centrifuged at 13,600 g and 4°C for 10 min. Two hundred microliters of the resulting supernatant was transferred to HPLC vials, and 20 μL of supernatant was injected into an LC-18 (4.6×150 mm, 5 μm) reverse-phase analytical column (Agilent Technologies, CA, USA). Separation of chlorantraniliprole was achieved using the mobile phase of 70% acetonitrile+30% water with a 1.0 mL/min flow rate at 30°C. The change in absorbance at 260 nm was quantified by peak integration and measured according to a previously prepared standard curve (S5 Fig). Three replicate samples were analyzed. A heat-inactivated enzyme at 100°C for 10 min was used as the control.

### Isolation of cytoplasmic and nucleic RNA

Nuclear and cytoplasmic RNA isolation was performed using the Cytoplasmic & Nuclear RNA Purification Kit (Norgen Biotek Corp, Ontario, Canada) following the manufacturer’s protocols. Then, quantitative real-time PCR was used to measure the RNA levels. In summary, equivalent volumes of nuclear and cytoplasmic RNA were used to perform RT-PCR to generate the cDNA template. Nuclear and cytoplasmic enrichment ratios were calculated to display the final result [53]. *β-actin* mRNA served as the cytoplasmic control, and U6 was used as the nuclear control.

### miRNA target prediction and dual-luciferase reporter assay

Two miRNA target prediction software programs, miRanda (http://www.microrna.org/microrna/getDownloads.do) [62] and RNAhybrid (http://bibiserv.techfak.uni-bielefeld.de/rnahybrid/welcome.html) [63], were used with default parameters to predict miRNAs that potentially targeted the 3’ untranslated region (UTR) of the *GSTu1* transcript. The luciferase reporter plasmid was constructed by inserting the wild-type or mutated target sequences of *GSTu1* between the firefly luciferase ORF (Open Reading Frame) and SV40 poly (A) into the pmirGLO vector (Promega, Leiden, Netherlands). The mutated target sequences were synthesized using a Site-Directed Mutagenesis Kit (Beyotime, Nanjing, China) following the manufacturer’s instructions. For the luciferase assay, HEK293T cells were cultured in a 96-well plate and transfected with the reporter plasmids and miRNA agomir or NC agomir using a calcium phosphate cell transfection kit (Beyotime) following the manufacturer’s instructions. Each well contained 0.2 μg plasmid and 90 nM miRNA agomir. Luciferase assays were performed using the Dual-Glo Luciferase Assay System (Promega) at 24 h posttransfection. Normalized firefly luciferase activity (firefly luciferase activity/Renilla luciferase activity) was compared with activity in the control groups. For each transfection, luciferase activity was averaged from the results of six replicates.

### RIP

A Magna RIP Kit (Millipore, Germany) was used to perform the RIP assay following Ye et al. [64]. Approximately 10 third instars were collected and homogenized on ice in RIP lysis buffer. The lysates were centrifuged at 13,600 g for 10 min at 4°C, and the supernatant (100 μl) was incubated with 5 μg of RIP Ab+ Ago-1 antibody (Millipore) or normal mouse IgG (Millipore) beads for 12 h at 4°C. Then, the beads were washed with RIP wash buffer several times. Finally, RNAs were purified using TRIzol reagent (Invitrogen) and used for cDNA synthesis. The transcript enrichment ratio for the purified RNAs was then determined by qRT-PCR. Three biological replicates and three technical replicates were performed.

### FISH and RPA

An antisense nucleic acid detection probe (5’-AATCTCTTTCTGCGGATTCATCTGAGCATATCCCTCGGTGAGGTG-3’) designed to detect *GSTu1* was labeled with Cy3. The probe for detecting lnc-*GSTu1*-AS (5’-TATCAAGTTACGCATCCAAGATAACAGAGTTAGATAGGTAAGTAC-3’) was labeled with FAM (GefanBio, China). The random shuffled probe (5’-UUCUCCGAACGUGUCACGUU-3’) and the probe (5’-UUGUACUACACAAAAGUACUG-3’) were used as lncRNA and mRNA negative controls, respectively. For fluorescence *in situ* hybridization, midguts were fixed in 4% paraformaldehyde for 2 h. The tissue slices were treated with 0.2 M hydrochloric acid and proteinase K and then incubated with the mRNA or miRNA probes at 65°C for 48 h in the dark. The samples were washed in PBS five times (1 min each time) at room temperature. Fluorescence signals were analyzed, and images were recorded using a Nikon Eclipse Ci microscope (Tokio, Japan).

Total RNA was extracted from the midgut of *P*. *xylostella* using TRIzol reagent and then digested with DNase I to remove DNA contamination. The purified RNA was treated with RNase A (Takara) to digest the single-stranded RNAs in digestion buffer. Following RT-PCR, agarose gel analysis was performed to identify the binding ability and the protective effect of lnc-*GSTu1*-AS on GSTu1.

### Western blotting

Antibodies against GSTu1 were synthesized by Beijing Protein Innovation Co., Ltd. (Beijing, China). An antibody against β-actin (TransGen Biotech, Beijing, China) was used as an internal control. Total proteins of each sample were extracted using a Tissue Protein Extraction Kit (Cwbio, China) following the manufacturer’s protocol. The protein concentration was then determined using a BCA Protein Assay Kit (Cwbio, Beijing, China). Extracted total proteins were first separated by 10% sodium dodecyl sulfate-polyacrylamide gel electrophoresis (SDS-PAGE) and subsequently transferred onto 0.45 μm-thick polyvinylidene fluoride (PVDF) membranes (Millipore, Germany). Membranes were then blocked with 5% skim milk (Biotopped, China) for 2 h and subsequently incubated with specific antibodies overnight at 4°C. Subsequently, membranes were washed with phosphate-buffered saline with Tween-20 (PBST) and then incubated with goat anti-rabbit immunoglobulin G (dilution rate 1: 20,000) (CwBio, China) for 1 h. Finally, specific protein bands were analyzed using an enhanced ECL Western blot assay kit (CwBio, China). Quantitative analysis of the Western blot results was performed using the program Image J.

### Statistical analysis

Data were analyzed using the Student’s *t*-test to evaluate significant differences between the agomir or antagomir/dsRNA treatment and the control groups. One-way analysis of variation (ANOVA) followed by Tukey’s multiple comparisons was used to compare the relative luciferase activities of each transfection group and the relative expression of *GSTu1* and lnc-*GSTu1*-AS at different developmental stages or in different tissues. All statistical analyses were conducted using SPSS software version 20. A *P*-value < 0.05 was considered to be statistically significant. All primers used in this study are listed in S3 Table.

## Supporting information

S1 Fig Sequence analysis of GSTu1. A: G-site and H-site residues of GSTu1 in *P*. *xylostella*. B: Phylogenetic analysis of GSTu1 among *P*. *xylostella* and other related insects. The conserved G-site residues are marked by red triangles, and the substrate binding pockets (H-sites) are indicated by blue triangles. The putative GSTu1 amino acid sequences of *Aedes aegypti* (XP_021706303), *Culex quinquefasciatu* (XP_001851103.2), *Drosophila melanogaster* (XP_014766382), *Musca domestica* (XP_005183645), *Lucilia cuprina* (XP_023295552), *Bombus terrestris* (XP_012169538), *Habropoda laboriosa* (XP_017796904), *Melipona quadrifasciata* (KOX72227), *Neodiprion lecontei* (XP_015515049), *Tribolium castaneum* (XP_975048), *Anoplophora glabripennis* (XP_018564199), *Leptinotarsa decemlineata* (APX61055), *Locusta migratoria* (AHC08059), *Helicoverpa armigera* (XP_021191578), *Spodoptera litura* (XP_022825963), *P*. *xylostella*, *Amyelois transitella* (XP_013196444), *Chilo suppressalis* (RVE50143), *Bombyx mori* (NP_001108462), *Papilio machaon* (XP_014361114), *Papilio xuthus* (NP_001299563) and *Papilio polytes* (NP_001298693) downloaded from GenBank were used for the phylogenetic analysis.
(TIF)

S2 Fig The search for RNA-binding proteins using RNA binding protein immunoprecipitation (RIP). (TIF)

S3 Fig The miRNAs targeting *GSTu1* predicted by miRanda and RNAhybrid. (TIF)

S4 Fig A schematic representation of all injection experiments. The upward and down arrows indicate that the expressions are up-regulated and down-regulated, respectively; The arrow with flat end indicates that the expression is unchanged.
(TIF)

S5 Fig The standard curve used for quantification of chlorantraniliprole. (TIF)

S1 Table Toxicity of chlorantraniliprole to different populations of *Plutella xylostella*. (XLSX)

S2 Table Sequences of miR-8525-5p and miR-8530-5p agomir and antagomir. (XLSX)

S3 Table All primers used in this study. (XLSX)
