# Supplementary figures and images for: Regulation of GSTu1-mediated insecticide resistance in Plutella xylostella by miRNA and lncRNA
Source: PLoS Genet. 2021 Oct 28;17(10):e1009888. doi: 10.1371/journal.pgen.1009888 (PMC8589219; doi:10.1371/journal.pgen.1009888)

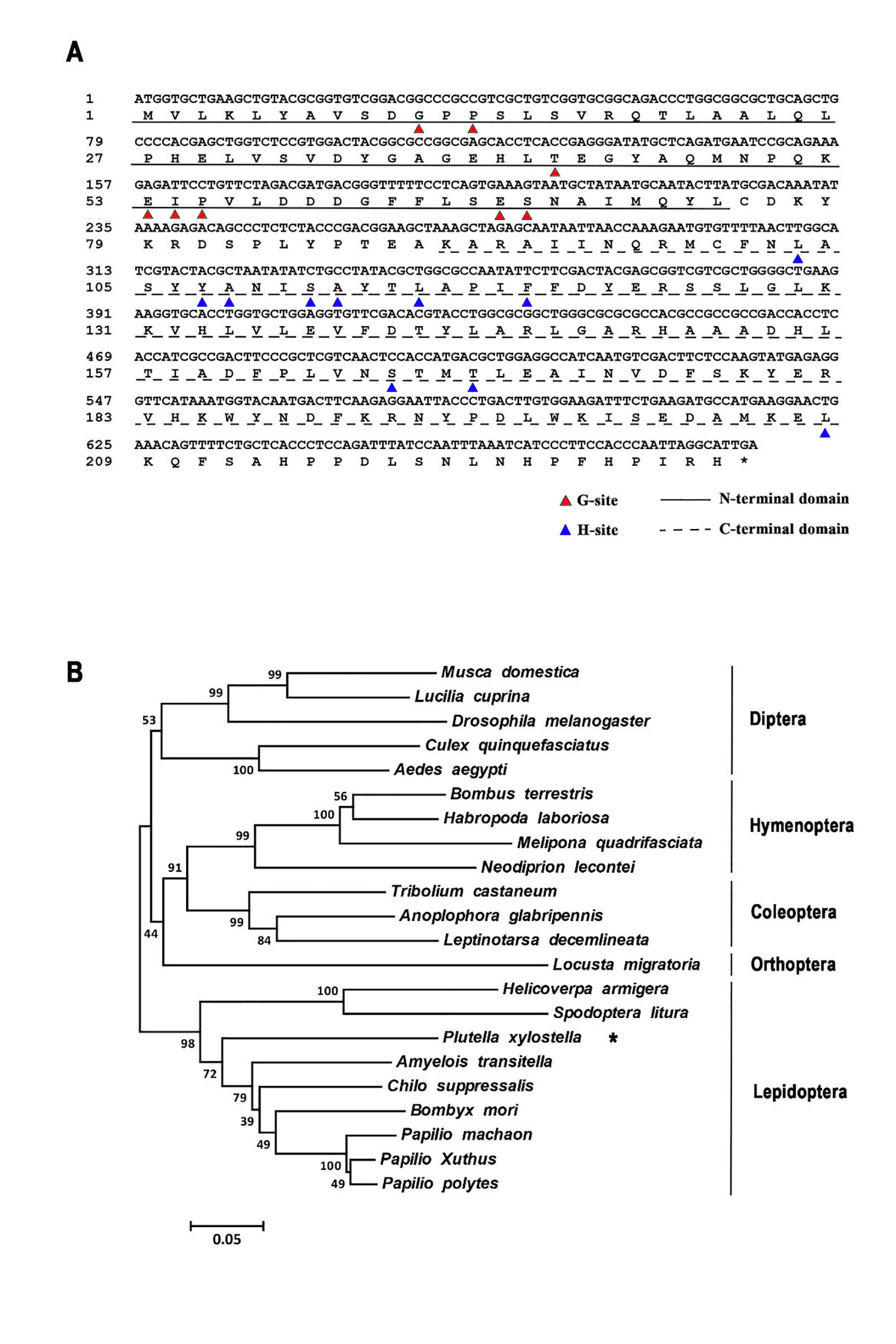

Supplement: S1 Fig — A: G-site and H-site residues of GSTu1 in P. xylostella. B: Phylogenetic analysis of GSTu1 among P. xylostella and other related insects. The conserved G-site residues are marked by red triangles, and the substrate binding pockets (H-sites) are indicated by blue triangles. The putative GSTu1 amino acid sequences of Aedes aegypti (XP_021706303), Culex quinquefasciatu (XP_001851103.2), Drosophila melanogaster (XP_014766382), Musca domestica (XP_005183645), Lucilia cuprina (XP_023295552), Bombus terrestris (XP_012169538), Habropoda laboriosa (XP_017796904), Melipona quadrifasciata (KOX72227), Neodiprion lecontei (XP_015515049), Tribolium castaneum (XP_975048), Anoplophora glabripennis (XP_018564199), Leptinotarsa decemlineata (APX61055), Locusta migratoria (AHC08059), Helicoverpa armigera (XP_021191578), Spodoptera litura (XP_022825963), P. xylostella, Amyelois transitella (XP_013196444), Chilo suppressalis (RVE50143), Bombyx mori (NP_001108462), Papilio machaon (XP_014361114), Papilio xuthus (NP_001299563) and Papilio polytes (NP_001298693) downloaded from GenBank were used for the phylogenetic analysis. (TIF) [file pgen.1009888.s001.tif]

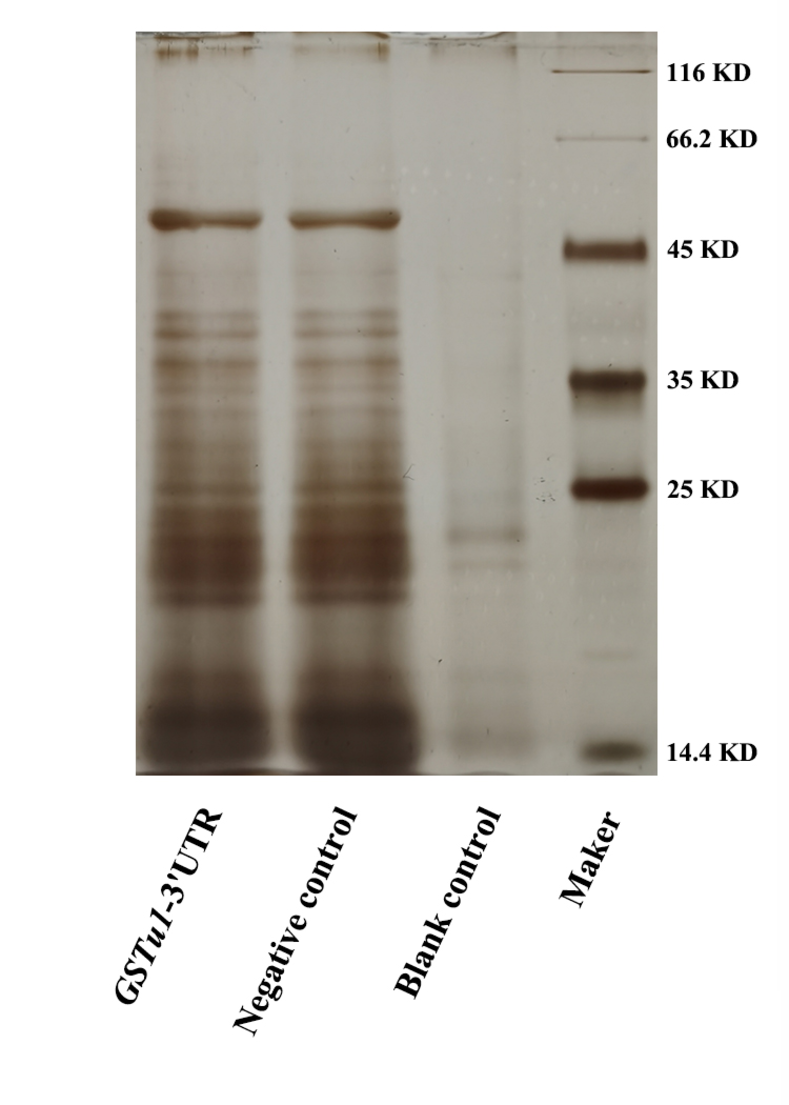

Supplement: S2 Fig — (TIF) [file pgen.1009888.s002.tif]

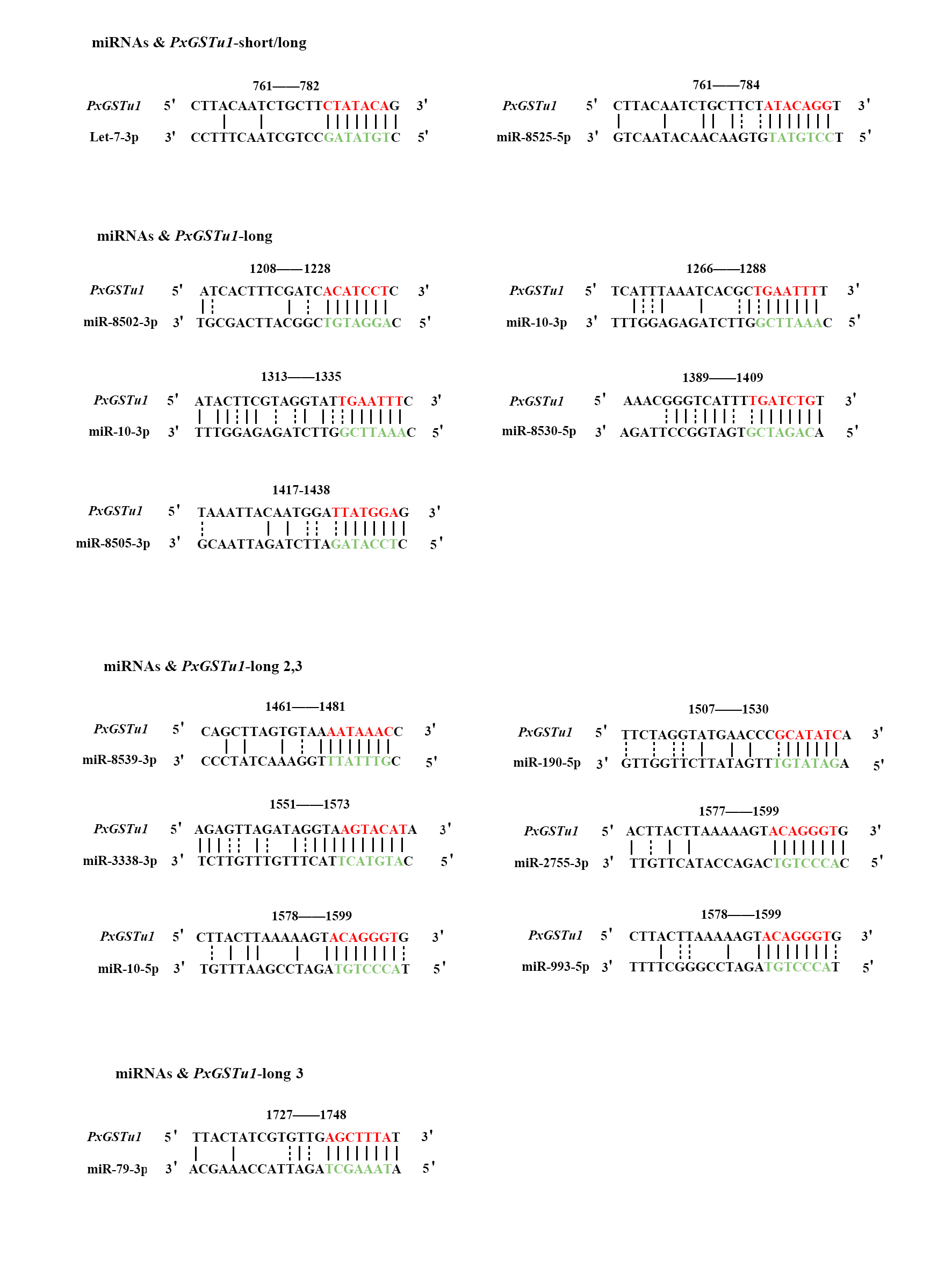

Supplement: S3 Fig — (TIF) [file pgen.1009888.s003.tif]

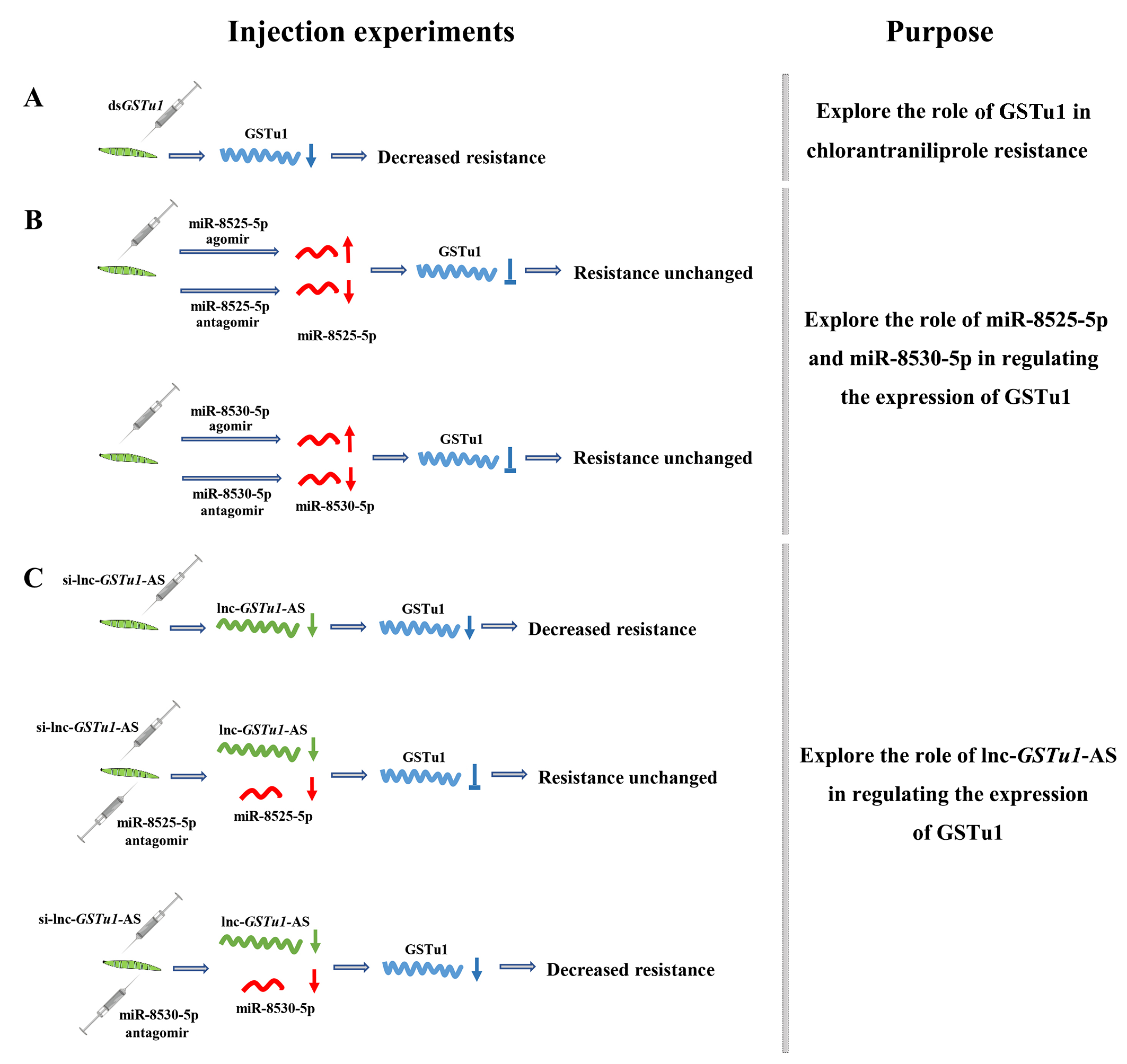

Supplement: S4 Fig — The upward and down arrows indicate that the expressions are up-regulated and down-regulated, respectively; The arrow with flat end indicates that the expression is unchanged. (TIF) [file pgen.1009888.s004.tif]

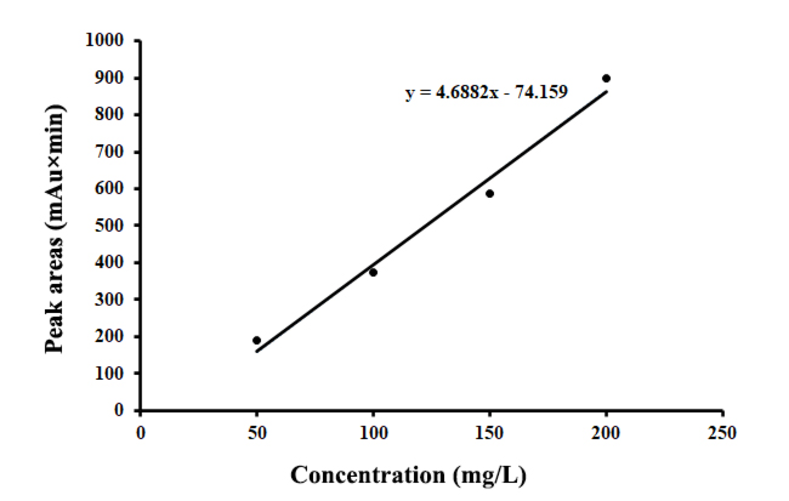

Supplement: S5 Fig — (TIF) [file pgen.1009888.s005.tif]
